# One-Pot Synthesis of Novel 2-Imino-5-Arylidine-Thiazolidine Analogues and Evaluation of Their Anti-Proliferative Activity against MCF7 Breast Cancer Cell Line

**DOI:** 10.3390/molecules27030841

**Published:** 2022-01-27

**Authors:** Marian N. Aziz, Arzoo Patel, Amany Iskander, Avisankar Chini, Delphine Gout, Subhrangsu S. Mandal, Carl J. Lovely

**Affiliations:** 1 Department of Chemistry and Biochemistry, University of Texas-Arlington, Arlington, TX 76019-0065, USA; mariannadyaziza.awadalla@uta.edu (M.N.A.); arzoo.patel@mavs.uta.edu (A.P.); amany.iskander@uta.edu (A.I.); avisankar.chini@mavs.uta.edu (A.C.); delphine.gout@uta.edu (D.G.); smandal@uta.edu (S.S.M.); 2 Department of Pesticide Chemistry, National Research Centre, Dokki, Giza 12622, Egypt

**Keywords:** hydrothiolation, propargyl amine, surface-mediated cyclization, cytotoxicity, X-ray

## Abstract

An efficient surface-mediated synthetic method to facilitate access to a novel class of thiazolidines is described. The rationale behind the design of the targeted thiazolidines was to prepare stable thiazolidine analogues and evaluate their anti-proliferative activity against a breast cancer cell line (MCF7). Most of the synthesized analogues exhibited increased potency ranging from 2–15-fold higher compared to the standard reference, cisplatin. The most active thiazolidines contain a halogenated or electron withdrawing group attached to the *N*-phenyl ring of exocyclic 2-imino group. However, combination of the two substituents did not enhance the activity. The anti-proliferative activity was measured in terms of IC_50_ values using an MTT assay.

## 1. Introduction

**Figure 1:**
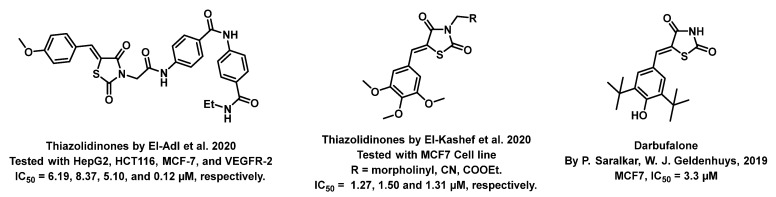
Recently published thiazolidinones targeting breast cancer.

**Figure 2:**
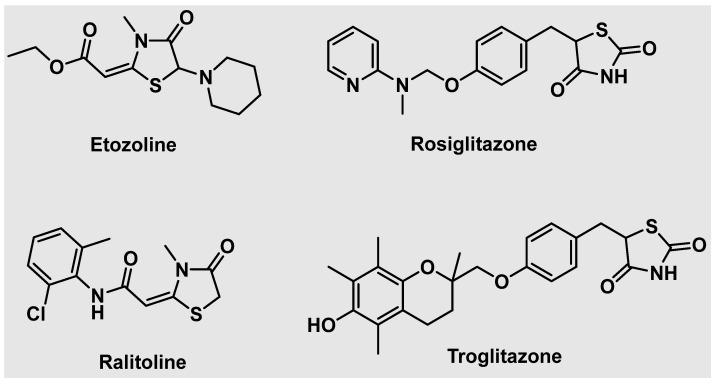
Approved drugs containing the thiazolidine core structure.

Thiazolidines, five-membered nitrogen- and sulfur-containing ring compounds, are among the most eminent of heterocyclic classes due to their broad applications. Intriguing chemical applications of thiazolidine derivatives include site-specific peptide and protein modification and chemical protein synthesis [1,2,3]. In addition, thiazolidines have been reported as potent agents against a number of ailments, and these include diabetes, epilepsy, viral and bacterial diseases, and cancer, to name a few [4]. Cancer is considered the second leading cause of death worldwide with a high mortality rate, exceeding more than 10 million annually [5]. Breast cancer is among the most feared of women’s diseases due to the fact there are multiple genotypes and phenotypes and that it is a metastatic and heterogenous solid tumor [6]. Therefore, there are a number of published studies focused on developing novel lead/hit compounds containing thiazolidines targeting cancers [7,8,9,10,11,12,13]. Figure 1 shows three different compounds described in recently published studies, in which novel thiazolidine analogues are reported as anti-proliferative agents targeting breast cancer [14,15,16]. Most of these derivatives contain 4-methoxy benzylidene derivatives and show good selectivity toward breast cancer cell lines. In addition, Figure 2 depicts four examples of marketed drugs containing a thiazolidine core structure, etozoline, troglitazone, rosiglitazone, and ralitoline. Etozoline is used as an antihypertensive and diuretic drug [17,18], while troglitazone and rosiglitazone are used as remedies for type 2 diabetes mellitus via enhancing PPARγ activity [19,20,21,22]. In addition, (*E*)-*N*-(2-chloro-6-methylphenyl)-2-(3-methyl-4-oxothiazolidin-2-ylidene)acetamide (ralitoline) is used as an anticonvulsant-thiazolidinone drug [23,24]. Therefore, thiazolidines exhibit a broad array of medicinal applications, which have inspired researchers to develop novel methodologies for their synthesis. Acid- and base-catalyzed thiazolidine syntheses have been reported as approaches to these heterocycles however, most of these synthetic methods require elevated temperatures and long reaction times and are characterized by low yields and moderate substrate scope [25,26,27,28,29]. In addition, ionic-liquid-assisted synthesis of thiazolidine is considered to be an efficient method but in some cases requires introduction of additional catalysts; otherwise, yields can be an issue [30]. Recently, nanoparticles have been used as catalysts because of their high surface-to-volume ratio that enhances the reactivity and selectivity of the reaction, especially in nano-heterogenous catalysts. All reported nanoparticles used in thiazolidine synthesis are reusable, but challenges remain, with the scope limited to aryl derivatives [31,32,33] and long reaction times [32,34]. In addition to these promising methodologies, multi-step reactions, metal catalysis, ultrasonic irradiation, microwave conditions, and catalyst-free and solvent-free approaches have been described for the synthesis of various derivatives of thiazolidines [4].

**Scheme 1:**
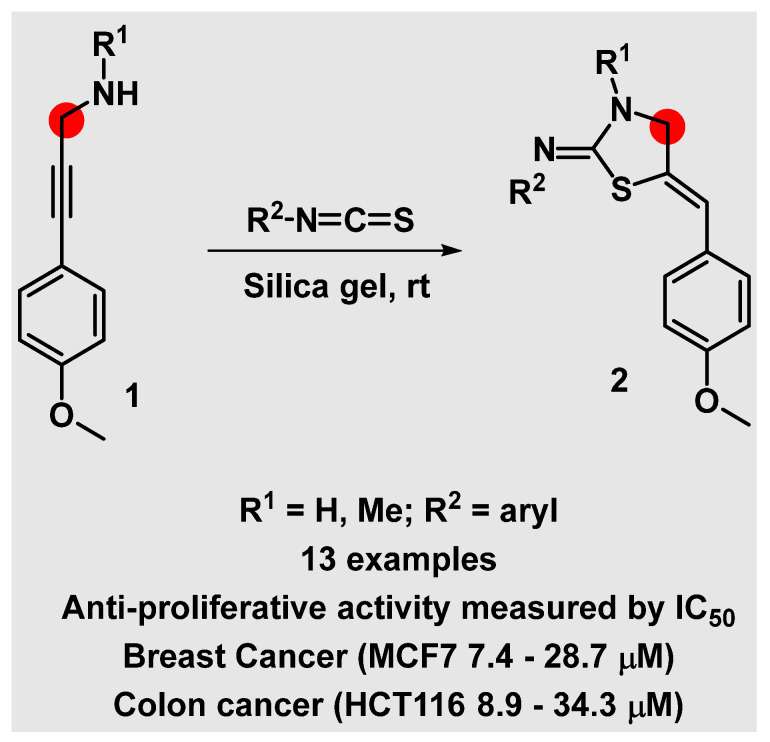
Previously synthesized and screened thiazolidines against breast and colon cancers.

A previously reported on-surface synthesis has been developed for thiazolidinones but it requires long reaction times and dichloromethane as solvent [35]. While, in our earlier work, silica gel was investigated and shown to promote fast cyclization of thioureas formed in situ from propargyl amines and isothiocyanates. Therefore, we have focused on developing this interesting one-pot chemistry further to access bio-active thiazolidine-based structures easily [36]. In 2019, our group published the synthesis of a novel group of thiazolidines using this procedure, some of which were prone to oxidation at C4 upon extended exposure to the ambient atmosphere producing thiazolidinones (Scheme 1) [13]. Most of the reported compounds showed good anti-tumor activity against colon and breast cancers (in vitro study using HCT116 and MCF7 cell lines), which inspired us to design new analogs belonging to thiazolidines system only. Therefore, in this present study, an efficient, fast, and convenient on-surface methodology is explored toward synthesis of 5-substituted thiazolidines in a one-pot reaction from propargyl amines and isothiocyanates. Since our previously prepared thiazolidines were more active against breast cancer in general, the anti-proliferative activity of the current new analogues against a breast cancer cell line (MCF7) is also described.

## 2. Results and Discussion

### 2.1. Chemistry

Initially, it was thought that blocking the oxidation hot spot in the thiazolidine structure, specifically the C4 methylene group, would mitigate the oxidation process. Therefore, we have designed new derivatives incorporating a methyl group at the C4 position and have evaluated the stability and antiproliferative activity of these derivatives. Thiazolidines **5a–l** were synthesized via reaction of propargyl amine **4** and various aryl isothiocyanates on silica gel at room temperature overnight.

**Scheme 2:**
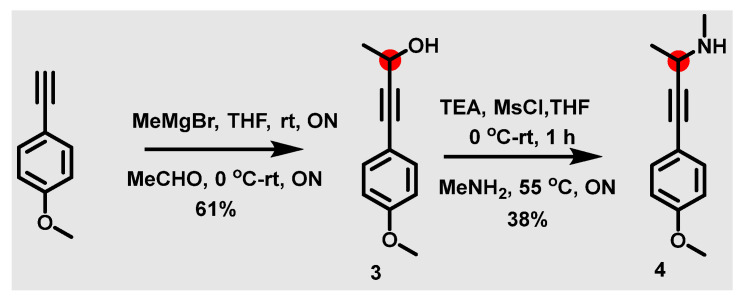
Synthesis of the targeted 4-(4-methoxyphenyl)-*N*-methylbut-3-yn-2-amine. ON, overnight.

Initially, several unsuccessful experiments were performed evaluating approaches for the synthesis of the targeted propargyl amine, including a three component reaction (3CR) of 4-ethynlyanisole, acetaldehyde, and methylamine catalyzed by CuI and/or CuBr [37,38,39]. Such conditions have worked well for us and others for the assembly of propargyl amines in other contexts. In addition, palladium catalysts for C-N bond formation have been used in 3CR under different conditions, but these were not successful either [40]. Therefore, the approach was modified, and propargyl alcohol **3** was synthesized first in good yield (61%) using a Grignard reaction from commercially available reagents (4-ethynylanisole, ethylmagnesium bromide and acetaldehyde). The propargyl amine **4** was prepared via an S_N_2 reaction by activating the alcohol first with methanesulfonyl chloride and then treating the corresponding sulfonate with methylamine in the presence of triethylamine as a base (Scheme 2).

**Scheme 3:**
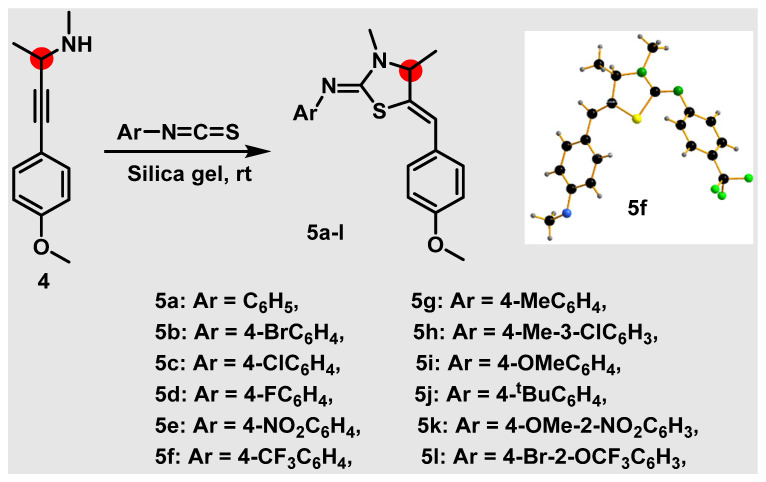
Synthesis of the fully substituted thiazolidines **5a**–**l**.

An initial negative result was obtained by treating the synthesized propargyl amine with phenyl isothiocyanate in dichloromethane solution at room temperature for 12 h. Adding silica gel as a solid support makes a significant difference by bringing the two reagents together on its surface presumably through hydrogen bonding interactions with the hydroxyl groups on its surface. Therefore, silica gel helps in thiourea formation and mediates and/or catalyzes the cyclization process through these types of interactions. The fully substituted thiazolidines were synthesized through mixing 4-(4-methoxyphenyl)-*N*-methylbut-3-yn-2-amine (**4**) and different aryl isothiocyanates (**5a–l**) in the presence of silica gel at room temperature with stirring overnight (Scheme 3). Solid starting materials were dissolved in a small amount of dichloromethane and then added to the silica gel with stirring to evaporate the solvent. The reaction was monitored by TLC by taking tiny amounts of silica gel reaction mixture and adding a few drops of dichloromethane. All of the isolated thiazolidines were purified by silica gel chromatography, and the structures were confirmed by spectroscopic analysis (IR, ^1^H NMR, ^13^C NMR, HRMS).

Analysis of compound **5f** via X-ray single crystal shows the constitution is as expected and that the geometry of the two exocyclic double bonds is exhibiting the same geometry of the previous reported thiazolidines, *Z*, *Z*’-configurations [13]. Colorless needle crystals of compound **5f** crystallized in a monoclinic space group P2_1_/c with four molecules per unit cell. Further details about X-ray single crystal study of **5f** is shown in the supplementary information file and experimental section. In addition, the ^1^H NMR spectrum shows long-range coupling between the C4-methyl group (at the hot spot) and the vinylic proton also confirming the *Z*-configuration for the exocyclic double bond. The vinylic proton and carbon for compound **5f** appear at δ_H_ = 6.49 ppm and δ_C_ = 119.5 ppm, respectively.

The initial rationale behind developing this chemistry was to investigate the bioactivity of different derivatives of thiazolidine, especially against breast cancer, and ultimately discovering new hits/leads to serve as anti-tumor agents. Although we have blocked the reactive methylene group using a methyl group, all the synthesized compounds were unstable, decomposing after extended periods at room temperature, and they were stored at −10 °C under an atmosphere of argon. TLC analysis showed formation of multiple components after leaving the molecules in deuterated chloroform or dichloromethane and exposure to the atmospheric air over the course of several days. Attempted purification of these mixtures by preparative TLC of the decomposed samples failed; however, while the isolated components appeared homogenous by TLC, we were unable to identify the decomposed product. An ^1^H NMR study of the reaction mixtures of these new derivatives after long-term exposure to air was not helpful due to formation of unidentifiable peaks. The instability of our previously published thiazolidines was determined by isolation of the oxidized thiazolidinones. Our assumption is that hydroxylation occurs at C4, and the resulting carbinolamine undergoes ring-opening and further oxidation. Therefore, samples for testing were synthesized immediately prior to their use in the MTT assays using breast cancer cell line (MCF7) to test their cytotoxicity with tumorous cell line. Compound **5g** has been excluded from biological screening due to its instability at –10 °C. A year later, simple TLC experiments were performed for all the derivatives stored in the freezer under argon, and they showed only one spot, referring to successful storage method for such unstable derivatives except **5g**. In addition, samples stored in DMSO at 4 °C exhibited good stability after a year especially compounds **5d** and **5e** which contain an electronic withdrawing group (F and NO_2_).

### 2.2. Anti-Proliferative Activity

**Figure 3:**
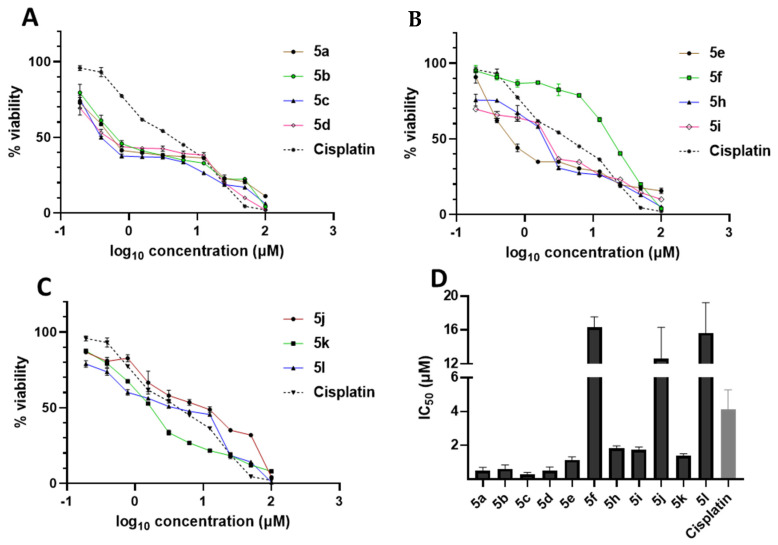
Anti-proliferative activity of thiazolidines **5a**–**f** and **5h**–**l**; (**A**–**C**) cell viability of MCF7 cell incubated with thiazolidine and tested by MTT assay after 96 h. (**D**) calculated IC_50_ based on non-linear data obtained from the cell viability study.

Evaluation of the biological activity of the targeted thiazolidines was the main motivation toward the synthesis and developing such interesting chemistry. Therefore, the synthesized compounds were tested with a breast cancer cell line (MCF7) using the MTT colorimetric assay using solutions of freshly prepared thiazolidines **5a–f** and **5h–l**. Cisplatin was used as a standard reference for the MTT obtained data. Figure 3A–C shows the cell viability using ten different concentrations of thiazolidines and cisplatin, and all the compounds exhibited activity in a dose-dependent manner. Figure 3D shows the cytotoxicity of the thiazolidines represented as IC_50_ values which express the concentration required from each compound to inhibit the growth of the MCF7 cells by 50%. Figure 3A depicts the effect of compounds **5a**–**d**, all of them behave similarly and showing more potency compared to cisplatin activity. Figure 3B,C show that most of the compounds are more active compared to cisplatin except for one compound in each graph, **5f** and **5j**. The bar graph (Figure 3D) indicates that most of synthesized compounds are more potent relative to the standard reference based on the calculated values of the corresponding IC_50_. Regarding the structure activity relationship, generally, all the synthesized compounds have a common scaffold of the thiazolidine core and 4-methoxybenzylidene group, but different substituents attached to exocyclic aryl amine. All the derivatives substituted with an electron withdrawing group exhibit higher potency (IC_50_ values) against MCF7 breast cancer cell lines compared to cisplatin. For instance, compounds **5a**–**e** exhibit the highest anti-proliferative activity among this library against MCF7 (IC_50_ = 0.50, 0.62, 0.27, 0.50, and 1.15 μM for **5a–e** respectively, Table 1) in comparison to the corresponding 4.14 μM observed for cisplatin. Compound **5a** with an unsubstituted phenyl ring on the exocyclic 2-imino group and thiazolidine **5d** *p*-fluorophenyl ring exhibit similar IC_50_ values (0.50 μM), displaying more than 8-fold potency. Replacing the fluoro group attached to exocyclic 2-imino group with a chloro group (**5c**) enhances the activity and increases the potency from 8 to 15- fold higher than cisplatin. Meanwhile, using a bromo group (**5b**) instead of a fluoro or chloro group does not show any improvement in the corresponding IC_50_, and yet the activity is still better than cisplatin (Table 1, entry 2 and 13). (*Z*)-*N*-(4-chlorophenyl)-5-((*Z*)-4-methoxybenzylidene)-3,4-dimethylthiazolidin-2-imine (**5c**) is the most potent compound among the screened series of thiazolidines, exhibiting higher than 15-fold anti-proliferative activity compared to the positive control. The electron donating groups attached to both phenyl rings (methoxy groups, compound **5i**) exhibited good activity higher than two-fold, while trifluoromethyl and *tert*-butyl groups attached to phenyl ring, compounds **5f** and **5j**, lowering the activity by three-fold compared with cisplatin (16.32 and 12.61 μM respectively). A compound bearing both an electron donating group with electron withdrawing groups on the same phenyl ring lowers the activity compared to the corresponding analogues with only electron withdrawing groups. A clear example is compound **5l** in which the bromo group does not affect the influence of the trifluoromethoxy group; thus, the corresponding IC_50_ value is 15.64 μM and the halogen (Br) group did not improve the activity in comparison to compound **5b** (IC_50_ = 0.62 μM). Combination of a methoxy and a nitro group in compound **5k** did not cause a significant effect on the corresponding potency (1.38 μM), which is still close to a single methoxy substituted phenyl group (compound **5i** with IC_50_ = 1.75 μM) and nitro group (compound **5e** with IC_50_ = 1.15 μM). The activity of the current library of thiazolidines is not only consistent with our previously published series, but it shows a good improvement in terms of IC_50_ values [13]. IC_50′_s of the previously synthesized thiazolidines ranged from 7.4–28.7 µM (Scheme 1), while the new derivatives ranged from 0.27–16.32 µM (Table 1). Moreover, the present thiazolidine library appears to be one of the most active thiazolidines against breast cancer cell (MCF7). In comparison to other published data, our new library revealed excellent activity in terms of IC_50_ compared to the three studies shown in Figure 1. This figure presents the most active three examples derived from these three different studies, IC_50_ values ranged from 3.3 to 5.1 µM. Therefore, our group is interested in developing such types of clean chemistry toward stable bio-active thiazolidines. Another novel and chemically stable series of thiazolidines is being prepared for publication in the near future with interesting anti-tumor activity.

## 3. Materials and Methods

### 3.1. Chemistry

All reagents were purchased from commercial suppliers and were used as received, unless otherwise indicated. Reactions were performed in oven-dried glassware (24 h at 120 °C) under an atmosphere of dry nitrogen using solvents that had been dried. Analytical thin layer chromatography (TLC) was performed on silica gel 60F254 aluminum precoated plates (0.25 mm layer) and were visualized using UV light at 254 nm. All chromatographic purifications were performed using the flash chromatography method on silica gel (200–400 mesh). The solvents were evaporated using (Rotavapor) rotary evaporation. ^1^H and ^13^C NMR (δ in ppm) spectra were recorded in CDCl_3_ (unless otherwise noted) at 500 and 125.8 MHz, respectively (unless otherwise noted). Residual CHCl_3_ (δ = 7.26) as reference for ^1^H NMR and carbon absorption of CDCl_3_ (δ = 77.0) as internal reference for ^13^C NMR were used. Data are reported as s, singlet; d, doublet; t, triplet; q, quartet; dd, doublet of doublets; dt, doublet of triplets; td, triplet of doublets; tt, triplet of triplets; m, multiplet. Infrared (IR) spectra were recorded neat samples using an ATR instrument. High resolution mass spectra (HR-MS) were acquired in the Shimadzu Center for Advanced Analytical Chemistry using electrospray ionization with mass being measured using electrospray ionization (ESI-TOF).

### 3.2. Procedures for the Synthesis of the Propargyl Alcohol and Amine:

Synthesis of 4-(4-methoxyphenyl)but-3-yn-2-ol (**3**):


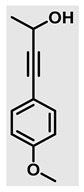


To a 500 mL flask containing 120 mL THF, 1-ethynyl-4-methoxybenzene (10.58 mL, 80 mmol, 1.0 equiv) was added while stirring at rt under argon. Then, EtMgBr (26.1 mL, 80 mmol, 1.0 equiv) was added, and the mixture was stirred under argon overnight. Acetaldehyde was then added (6.81 mL, 120 mmol, 1.5 equiv) while stirring at 0 °C forming a golden yellow solution. Then, the solution was warmed to rt and stirring continued overnight. The reaction was quenched with saturated aqueous NaHCO_3_ (30 mL) and then extracted with ethyl acetate (3 × 50 mL). The combined organic extracts were washed with brine solution (50 mL) then dried over sodium sulfate. The solvent was evaporated, and the crude product was purified by the flash column chromatography using 0–20% ethyl acetate/hexanes to afford the desired alcohol in good yield (9.5 g, 63%). ^1^H NMR (500 MHz, CDCl_3_) δ 7.33–7.22 (m, 2H), 6.80–6.69 (m, 2H), 4.67 (qd, *J* = 6.6, 1.2 Hz, 1H), 3.73 (d, *J* = 1.3 Hz, 3H), 1.47 (dd, *J* = 6.6, 1.2 Hz, 3H). ^13^C NMR (126 MHz, CDCl_3_) δ 159.7, 133.2, 114.7, 113.9, 89.7, 83.9, 58.9, 55.3, 24.5. FT-IR (neat, cm^−1^): 3353, 2985, 2840, 2226, 1605, 1566, 1506, 1371, 1076, 835. HRMS (*m/z*): calc for C_11_H_13_O_2_ [M + H]^+^ 177.0910 found 177.0904.

Synthesis of 4-(4-methoxyphenyl)-N-methylbut-3-yn-2-amine (**4**):


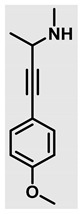


To a solution of 4-(4-methoxyphenyl)but-3-yn-2-ol (**3**) (0.34 g, 1.9 mmol, 1.0 equiv) in THF (5 mL, 2.5/mmol), triethylamine (1.32 mL, 9.5 mmol, 5.0 equiv) was added at rt then the mixture cooled in ice bath for 15 min. Then, methane sulfonyl chloride (0.29 mL, 3.8 mmol, 2.0 equiv) was added dropwise at 0 °C and the mixture was stirring for more 15 min then warmed up to the room temperature for 1 h. Methyl amine in THF solution (5.3 mL, 9.5 mmol, 5.0 equiv) was added and the mixture was heated up to 50 °C and allowed to stir for 24 h. The reaction mixture was concentrated, and then, the gummy material was dissolved in ethyl acetate (30 mL) and washed with aqueous NaHCO_3_ (20 mL). The aqueous layer was extracted with CH_2_Cl_2_ (3 × 30 mL) then the combined organic extracts were dried over sodium sulfate. The solvent was removed by rotary evaporation and then the crude product was purified by flash column chromatography using 7% methanol in dichloromethane to afford the desired amine in a modest yield (0.12 g, 34%). ^1^H NMR (500 MHz, CDCl_3_) δ 7.38–7.29 (m, 2H), 6.86–6.74 (m, 2H), 3.78 (s, 3H), 3.66 (q, *J* = 6.8 Hz, 1H), 2.78 (s, 1H), 2.55 (s, 3H), 1.44 (d, *J* = 6.8 Hz, 3H). ^13^C NMR (126 MHz, CDCl_3_) δ 159.5, 133.2, 115.3, 113.9, 89.2, 83.4, 55.3, 47.5, 33.7, 22.0. FT-IR (neat, cm^−1^): 2962, 2934, 2835, 2767, 2458, 2229, 1603, 1507, 1290, 1243, 1026, 832. HRMS (*m/z*): calc for C_12_H_16_ON [M + H]^+^ 190.1226 found 190.1228.

General procedure for the synthesis of thiazolidine derivatives:

To a 50 mL RBF containing 1.0 g of silica gel, 4-(4-methoxyphenyl)-*N*-methylbut-3-yn-2-amine (**4**) (1.0 equiv, 0.5–0.7 mmol) and the corresponding isothiourea derivative (1.0 equiv, 0.5–0.7 mmol) was added while stirring. The resulting slurry was placed under an argon atmosphere while stirring was continued at rt overnight. The silica gel containing the reaction mixture was poured directly onto a packed silica gel column followed elution with the appropriate solvent mixtures to afford the desired thiazolidines in good yield.


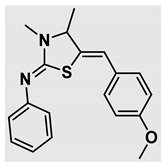


(Z)-5-((Z)-4-methoxybenzylidene)-3,4-dimethyl-N-phenylthiazolidin-2-imine (**5a**): Obtained from reaction of 4-(4-methoxyphenyl)-*N*-methylbut-3-yn-2-amine (0.54 mmol, 103 mg) and phenyl isothiocyanate (0.54 mmol, 75.3 mg) as a semi-solid yellow material (34.0 mg, 60%), purified by chromatography (25% EtOAc/Hexanes). ^1^H NMR (500 MHz, CDCl_3_) δ 7.36 (t, *J* = 7.6 Hz, 2H), 7.26 (d, *J* = 8.5 Hz, 2H), 7.13 (td, *J* = 7.4, 1.2 Hz, 1H), 7.08–7.03 (m, 2H), 6.95–6.84 (m, 2H), 6.50 (d, *J* = 1.6 Hz, 1H), 4.57 (qd, *J* = 6.3, 1.5 Hz, 1H), 3.84 (s, 3H), 3.18 (s, 3H), 1.58 (d, *J* = 6.3 Hz, 3H). ^13^CNMR (125 MHz, CDCl_3_) δ 158.6, 155.8, 151.3, 133.2, 132.5, 129.3, 129.0, 128.7, 123.3, 122.5, 119.6, 114.0, 114.0, 65.1, 55.3, 31.8, 21.0; FT-IR (neat, cm^−1^): 2959, 2920, 2850, 1614, 1584, 1508, 1247, 1176, 1027, 765; HRMS (*m/z*): calc for C_19_H_21_N_2_OS [M + H]^+^ 325.1369 found 325.1345.


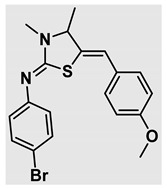


(Z)-N-(4-bromophenyl)-5-((Z)-4-methoxybenzylidene)-3,4-dimethylthiazolidin-2-imine (**5b**): Obtained from reaction of 4-(4-methoxyphenyl)-*N*-methylbut-3-yn-2-amine (0.73 mmol, 136 mg) and *p*-bromophenyl isothiocyanate (0.73 mmol, 160 mg) as a semi-solid yellow material (41.7 mg, 61%), purified by chromatography (20% EtOAc/Hexanes). ^1^H NMR (500 MHz, CDCl_3_) δ 7.43–7.33 (m, 2H), 7.23–7.14 (m, 2H), 6.87 (dt, *J* = 8.6, 1.8 Hz, 4H), 6.45 (d, *J* = 1.8 Hz, 1H), 4.52 (qt, *J* = 6.4, 1.8 Hz, 1H), 3.78 (d, *J* = 1.7 Hz, 3H), 3.11 (d, *J* = 1.7 Hz, 3H), 1.52 (dd, *J* = 6.3, 1.7 Hz, 3H). ^13^CNMR (125 MHz, CDCl_3_) δ 158.7, 132.0, 131.9, 129.3, 128.5, 124.4, 120.0, 116.2, 114.1, 65.3, 55.3, 31.8, 21.0; FT-IR (neat, cm^−1^): 2957, 2923, 2851, 2834, 1604, 1573, 1508, 1481, 1247, 1175, 1029, 858; HRMS (*m/z*): calc for C_19_H_20_N_2_OSBr [M + H]^+^ 403.0474 found 403.0451.


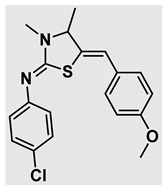


(*Z*)-*N*-(4-chlorophenyl)-5-((*Z*)-4-methoxybenzylidene)-3,4-dimethylthiazolidin-2-imine (**5c**): Obtained from reaction of 4-(4-methoxyphenyl)-*N*-methylbut-3-yn-2-amine (0.63 mmol, 118 mg) and *p*-chlorophenyl isothiocyanate (0.63 mmol, 108 mg) as a semi-solid yellow material (68.3 mg, 61%), purified by chromatography (25% EtOAc/Hexanes). ^1^H NMR (500 MHz, CDCl_3_) 7.33–7.15 (m, 4H), 7.00–6.82 (m, 4H), 6.46 (d, *J* = 2.1 Hz, 1H), 4.51 (dddt, *J* = 7.7, 6.6, 4.9, 1.4 Hz, 1H), 3.79 (d, *J* = 2.0 Hz, 3H), 3.1 (d, *J* = 2.0 Hz, 3H), 1.52 (dd, *J* = 6.5, 2.0 Hz, 3H). ^13^CNMR (125 MHz, CDCl_3_) δ 158.7, 156.1, 150.3, 132.2, 129.3, 129.1, 128.6, 128.3, 123.8, 119.9, 114.1, 65.2, 55.4, 31.7, 21.1. FT-IR (neat, cm^−1^): 2956, 2922, 2851, 1722, 1606, 1508, 1461, 1202, 832; HRMS (*m/z*): calc for C_19_H_20_N_2_OSCl [M + H]^+^ 359.0979 found 359.0946.


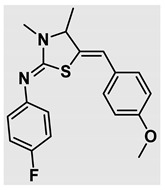


(*Z*)-*N*-(4-fluorophenyl)-5-((*Z*)-4-methoxybenzylidene)-3,4-dimethylthiazolidin-2-imine (**5d**): Obtained from reaction of 4-(4-methoxyphenyl)-*N*-methylbut-3-yn-2-amine (0.53 mmol, 100 mg) and *p*-fluorophenyl isothiocyanate (0.53 mmol, 80 mg) as a white solid (81.4 mg, 45%), m.p. 122–123 °C, purified by chromatography (25% EtOAc/Hexanes). ^1^H NMR (500 MHz, CDCl_3_) δ 7.24–7.14 (m, 2H), 7.02–6.90 (m, 4H), 6.88–6.82 (m, 2H), 6.46 (d, *J* = 1.8 Hz, 1H), 4.53 (qt, *J* = 6.4, 1.9 Hz, 1H), 3.79 (d, *J* = 2.0 Hz, 3H), 3.13 (d, *J* = 1.8 Hz, 3H), 1.53 (dd, *J* = 6.5, 2.0 Hz, 3H). ^13^C NMR (126 MHz, CDCl_3_) δ 158.7, 158.5, 132.0, 129.3, 128.5, 123.8, 123.7, 120.0, 115.7, 115.5, 114.1, 65.4, 55.3, 31.8, 21.0. FT-IR (neat, cm^−1^):2981, 2956, 2928, 2833, 1605, 1572, 1497, 1449, 1308, 1286, 1251, 1087, 1031, 861. HRMS (*m/z*): calc for C_19_H_20_N_2_OFS [M + H]^+^ 343.1275 found 343.1248.


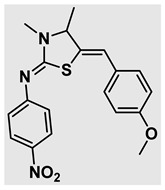


(*Z*)-5-((*Z*)-4-methoxybenzylidene)-3,4-dimethyl-*N*-(4-nitrophenyl)thiazolidin-2-imine (**5e**): Obtained from reaction of 4-(4-methoxyphenyl)-*N*-methylbut-3-yn-2-amine (0.56 mmol, 106 mg) and *p*-nitrophenyl isothiocyanate (0.56 mmol, 104 mg) as a yellow solid (57.0 mg, 95%), m.p. 120-122 C, purified by chromatography (25% EtOAc/Hexanes). ^1^H NMR (500 MHz, CDCl_3_) δ 8.20–8.13 (m, 2H), 7.21–7.15 (m, 2H), 7.08 (d, *J* = 8.5 Hz, 2H), 6.93–6.84 (m, 2H), 6.50 (d, *J* = 1.5 Hz, 1H), 4.63–4.55 (m, 1H), 3.79 (d, *J* = 1.4 Hz, 3H), 3.16 (s, 3H), 1.56 (dd, *J* = 6.3, 1.4 Hz, 3H). ^13^C NMR (126 MHz, CDCl_3_) δ 158.9, 129.3, 128.1, 125.1, 122.9, 120.8, 114.2, 65.5, 55.3, 31.9, 21.1. FT-IR (neat, cm^−1^):2981, 2958, 2921, 2833, 1615, 1576, 1507, 1392, 1284, 1214, 851; HRMS (*m/z*): calc for C_19_H_20_N_3_O_3_S [M + H]^+^ 370.1220 found 370.1196.


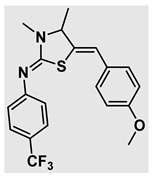


(*Z*)-5-((*Z*)-4-methoxybenzylidene)-3,4-dimethyl-*N*-(4-(trifluoromethyl)phenyl)thiazolidin-2-imine (**5f**): Obtained from reaction of 4-(4-methoxyphenyl)-*N*-methylbut-3-yn-2-amine (0.47 mmol, 89 mg) and *p*-(trifluoromethyl)phenyl isothiocyanate (0.47 mmol, 106 mg) as a pale yellow solid (33.0 mg, 58%), m.p. 120–121 °C, purified by chromatography (25% EtOAc/Hexanes). ^1^H NMR (500 MHz, CDCl_3_) δ 7.55 (dd, *J* = 8.6, 2.4 Hz, 2H), 7.18 (dd, *J* = 8.9, 2.5 Hz, 2H), 7.11 (d, *J* = 8.2 Hz, 2H), 6.90–6.86 (m, 2H), 6.49 (d, *J* = 2.3 Hz, 1H), 4.59 (d, *J* = 6.7 Hz, 1H), 3.86–3.73 (d, *J* = 3.5 Hz, 3H), 3.20 (s, 3H), 1.55 (dd, *J* = 6, 2.5 Hz, 3H). ^13^C NMR (126 MHz, CDCl_3_) δ 158.8, 156.1, 154.5, 131.8, 129.3, 128.5, 126.3, 126.3, 126.2, 126.2, 125.1, 123.7, 122.6, 120.1, 114.1, 65.2, 55.3, 31.7, 21.1.; FT-IR (neat, cm^−1^): 2958, 2925, 2851, 2838, 1625, 1590, 1509, 1318, 1250, 1101, 1062, 846; HRMS (*m/z*): calc for C_20_H_20_N_2_OF_3_S [M + H]^+^ 393.1243 found 393.1220.


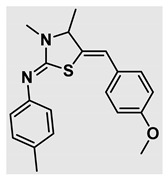


(*Z*)-5-((*Z*)-4-methoxybenzylidene)-3,4-dimethyl-*N*-(*p*-tolyl)thiazolidin-2-imine (**5g**): Obtained from reaction of 4-(4-methoxyphenyl)-*N*-methylbut-3-yn-2-amine (0.53 mmol, 100 mg) and *p*-tert-butylphenyl isothiocyanate (0.53 mmol, 75 mg) as a pale yellow solid (35 mg, 20 %), m.p. 110–111 °C, purified by chromatography (25% EtOAc/Hexanes). ^1^H NMR (500 MHz, CDCl_3_) δ 7.32–7.24 (m, 2H), 7.22–7.16 (m, 2H), 6.93–6.78 (m, 3H), 6.41 (d, *J* = 1.7 Hz, 1H), 4.50–4.42 (m, 1H), 3.75 (d, *J* = 1.0 Hz, 3H), 3.08 (d, *J* = 1.1 Hz, 3H), 1.55–1.44 (m, 3H), 1.31 (s, 3H).^13^C NMR (126 MHz, CDCl_3_) δ 158.7, 156.2, 150.5, 134.3, 132.3, 131.2, 130.4, 129.4, 128.6, 123.0, 120.8, 119.9, 114.1, 65.2, 55.4, 31.7, 21.1, 19.6. FT-IR (neat, cm^−1^): 2935, 2914, 2855, 1593, 1509, 1355, 1243, 1179, 1129, 838. HRMS (*m/z*): calc for C_20_H_23_N_2_OS [M + H]^+^ 339.1515 found 339.1514.


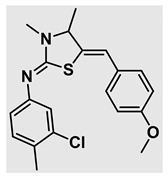


(*Z*)-*N*-(3-chloro-4-methylphenyl)-5-((*Z*)-4-methoxybenzylidene)-3,4-dimethylthiazolidin-2-imine (**5h**): Obtained from reaction of 4-(4-methoxyphenyl)-*N*-methylbut-3-yn-2-amine (0.53 mmol, 100 mg) and 3-chloro-4-methylphenyl isothiocyanate (0.53 mmol, 89 mg) as a pale-yellow solid (90.6 mg, 46%), m.p. 109–111 °C, purified by chromatography (25% EtOAc/Hexanes). ^1^H NMR (500 MHz, CDCl_3_) δ 7.36–7.07 (m, 3H), 7.08–6.87 (m, 2H), 6.85 (dq, *J* = 7.7, 2.3 Hz, 1H), 6.50 (q, *J* = 1.9 Hz, 1H), 4.56 (qd, *J* = 6.2, 4.4 Hz, 1H), 3.93–3.78 (m, 3H), 3.15 (d, *J* = 4.0 Hz, 2H), 2.40 (d, *J* = 4.4 Hz, 3H), 1.57 (t, *J* = 5.6 Hz, 3H). ^13^C NMR (126 MHz, CDCl_3_) δ 158.7, 156.4, 150.1, 134.3, 132.1, 131.1, 130.5, 129.3, 128.5, 123.1, 120.8, 119.9, 114.1, 65.3, 55.3, 31.8, 21.1, 19.5; FT-IR (neat, cm^−1^): 2960, 2916, 2834, 1591, 1507, 1248, 1177, 1025, 866; HRMS (*m/z*): calc for C_20_H_22_N_2_OSCl [M + H]^+^ 373.1136 found 373.1115.


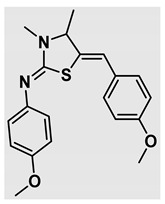


(*Z*)-5-((*Z*)-4-methoxybenzylidene)-*N*-(4-methoxyphenyl)-3,4-dimethylthiazolidin-2-imine (**5i**): Obtained from reaction of 4-(4-methoxyphenyl)-*N*-methylbut-3-yn-2-amine (0.59 mmol, 113 mg) and *p*-methoxyphenyl isothiocyanate (0.59 mmol, 100 mg) as a semi-solid yellow material (40.0 mg, 63%), purified by chromatography (25% EtOAc/Hexanes). ^1^H NMR (500 MHz, CDCl_3_) δ 7.23–7.17 (m, 2H), 6.95–6.88 (m, 2H), 6.85 (dd, *J* = 8.8, 3.5 Hz, 4H), 6.43 (d, *J* = 1.8 Hz, 1H), 4.50 (qd, *J* = 6.3, 1.7 Hz, 1H), 3.80 (s, 3H), 3.78 (s, 3H), 3.11 (s, 3H), 1.52 (d, *J* = 6.3 Hz, 3H). ^13^C NMR (126 MHz, CDCl_3_) δ 158.6, 156.3, 155.9, 132.6, 129.3, 128.7, 123.3, 119.6, 114.2, 114.0, 65.2, 55.5, 55.3, 31.8, 21.0. FT-IR (neat, cm^−1^): 3033, 2954, 2925, 2833, 1602, 1502, 1236, 1133, 1028, 830; HRMS (*m/z*): calc for C_20_H_23_N_2_O_2_S [M + H]^+^ 355.1475 found 355.1437.


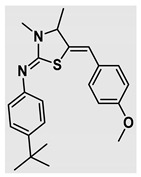


(*Z*)-*N*-(4-(tert-butyl)phenyl)-5-((*Z*)-4-methoxybenzylidene)-3,4-dimethylthiazolidin-2-imine (**5j**): Obtained from reaction of 4-(4-methoxyphenyl)-*N*-methylbut-3-yn-2-amine (0.47 mmol, 89 mg) and *p*-tert-butylphenyl isothiocyanate (0.47 mmol, 95 mg) as a pale yellow solid (27.4 mg, 95%), m.p. 122–123 °C, purified by chromatography (25% EtOAc/Hexanes). ^1^H NMR (500 MHz, CDCl_3_) δ 7.32 (d, *J* = 8.3 Hz, 2H), 7.19 (d, *J* = 8.4 Hz, 2H), 7.02 (d, *J* = 8.1 Hz, 2H), 6.87 (d, *J* = 8.5 Hz, 2H), 6.49 (s, 1H), 4.60 (d, *J* = 6.7 Hz, 1H), 3.79 (d, *J* = 1.0 Hz, 3H), 3.28 (s, 3H), 1.56 (d, *J* = 6.4 Hz, 3H), 1.32 (s, 9H). ^13^C NMR (126 MHz, CDCl_3_) δ 158.6, 155.2, 148.6, 145.8, 133.0, 129.3, 128.8, 125.9, 121.8, 119.5, 114.1, 64.9, 55.4, 34.4, 31.8, 31.6, 21.1.; FT-IR (neat, cm^−1^):2957, 2929, 2853, 2836, 1597, 1508, 1248, 1176, 837; HRMS (*m/z*): calc for C_23_H_29_N_2_OS [M + H]^+^ 381.1995 found 381.1967.


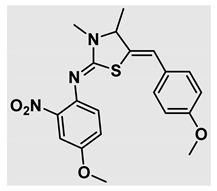


(*Z*)-*N*-(4-methoxy-2-nitrophenyl)-5-((*Z*)-4-methoxybenzylidene)-3,4-dimethylthiazolidin-2-imine (**5k**): Obtained from reaction of 4-(4-methoxyphenyl)-*N*-methylbut-3-yn-2-amine (0.53 mmol, 100 mg) and 4-methoxy-2-nitrophenyl isothiocyanate (0.53 mmol, 118 mg) as a semi-solid yellow material (43.0 mg, 63%), purified by chromatography (25% EtOAc/Hexanes). ^1^H NMR (500 MHz, CDCl_3_) δ 7.43 (t, *J* = 2.3 Hz, 1H), 7.16 (dd, *J* = 8.7, 2.0 Hz, 2H), 7.07 (ddd, *J* = 8.9, 3.0, 1.8 Hz, 1H), 7.00 (dd, *J* = 8.8, 1.8 Hz, 1H), 6.89–6.79 (m, 2H), 6.46 (d, *J* = 1.8 Hz, 1H), 4.58 (qt, *J* = 6.3, 1.8 Hz, 1H), 3.84 (d, *J* = 1.9 Hz, 3H), 3.78 (d, *J* = 1.9 Hz, 3H), 3.12 (d, *J* = 1.9 Hz, 3H), 1.55 (dd, *J* = 6.4, 1.9 Hz, 3H). ^13^C NMR (126 MHz, CDCl_3_) δ 158.7, 155.3, 131.6, 129.3, 128.4, 126.3, 121.0, 120.2, 114.1, 108.8, 65.8, 55.9, 55.3, 31.7, 21.2; FT-IR (neat, cm^−1^): 2965, 2929, 2836, 1604, 1508, 1247, 1176, 1029, 856; HRMS (*m/z*): calc for C_20_H_22_N_3_O_4_S [M + H]^+^ 400.1326 found 400.1302.


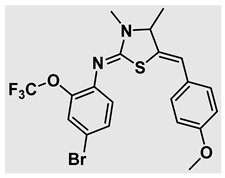


(*Z*)-*N*-(4-bromo-2-(trifluoromethoxy)phenyl)-5-((*Z*)-4-methoxybenzylidene)-3,4-dimethylthiazolidin-2-imine (**5l**): Obtained from reaction of 4-(4-methoxyphenyl)-*N*-methylbut-3-yn-2-amine (0.56 mmol, 106 mg) and 4-bromo-2-(trifluoromethoxy)phenyl isothiocyanate (0.56 mmol, 178 mg) as a semi-solid yellow material (47.0 mg, 57%), purified by chromatography (25% EtOAc/Hexanes). ^1^H NMR (500 MHz, CDCl_3_) δ 7.38 (dt, *J* = 2.5, 1.2 Hz, 1H), 7.34 (ddd, *J* = 8.5, 2.3, 1.2 Hz, 1H), 7.19 (d, *J* = 8.6 Hz, 2H), 6.95 (dd, *J* = 8.5, 1.1 Hz, 1H), 6.91–6.84 (m, 2H), 6.46 (d, *J* = 1.7 Hz, 1H), 4.59–4.52 (m, 1H), 3.79 (d, *J* = 1.1 Hz, 3H), 3.12 (d, *J* = 1.2 Hz, 3H), 1.53 (dd, *J* = 6.4, 1.2 Hz, 3H). ^13^C NMR (126 MHz, CDCl_3_) δ 158.8, 157.0, 143.6, 142.1, 131.5, 130.6, 129.3, 128.4, 125.5, 125.3, 121.6, 120.1, 115.1, 114.1, 65.6, 55.3, 31.6, 21.1; FT-IR (neat, cm^−1^): 2957, 2923, 2853, 1612, 1509, 1245, 1214, 1161, 1032, 937, 819; HRMS (*m/z*): calc for C_20_H_19_BrF_3_N_2_O_2_S [M + H]^+^ 487.0297 found 487.0307.

### 3.3. X-ray Single Crystal Study

The diffraction studies were carried out on a Bruker Kappa APEX-II CCD diffractometer at 100(2) K using monochromated Mo-Ka radiation (l = 0.71073 Å) and a detector-to-crystal distance of 5.220 cm. A 0.17 × 0.045 × 0.030 mm colorless needle was mounted on a Cryoloop with Nujol oil. Data were collected in a hemisphere or full sphere of reciprocal space with 0.3° scans in ω for an exposure time of 30 s per frame up to a maximum 2θ value of 56.66°. A total of 3759 reflections were collected covering the indices, −13 ≤ h ≤ 13, −29 ≤ k ≤ 28, −12 ≤ l ≤ 12. 2336 reflections were found to be symmetry independent. Indexing and unit cell refinement indicated a monoclinic lattice. The space group was found to be P21/c. The measured intensities were corrected for Lorenz and polarization effects and were further corrected for absorption using the multi-scan method SCALE3 ABSPACK. Based on the data, structural model was obtained by direct method using the Superflip subroutine implemented in the JANA2006 software package. The refinement was performed via full-matrix least-squares on F2 by using the JANA2006 software package. Crystallographic data are summarized in Table S1 (Supplementary Materials).

### 3.4. Anti-Proliferative Activity

Human breast cancer cells, MCF7, were grown and maintained in DMEM supplemented with 10% heat-inactivated FBS (fetal bovine serum), 2  mM L-glutamine, 100 units/mL penicillin and 0.1 mg/mL streptomycin, in a humidified incubator with 5% CO_2_. For the cytotoxicity analysis, MCF7 cells were seeded in a 96-well tissue culture-treated plate (~7000 cells/well, 200 μL volume). After 18 h, cells were treated with 0.19–100 μM (final concentration) and incubated for 96 h. Briefly, prior to the treatment, the old tissue culture media from the 96-well plate were discarded and replaced with 100 μL fresh media and then treated with 100 μL the diluted compound (in complete DMEM) to obtain respective desired final concentration of each compound. Stock solution (100 mM) of each compound was prepared in DMSO. After 96 h of incubation, 20 μL of freshly prepared MTT (thiazolyl blue tetrazolium bromide, 5 mg/mL stock in PBS) was added to each well and further incubated inside a cell culture incubator for an additional 4 h. The supernatant was carefully removed and discarded. Cells were washed once with 1X PBS. 100 μL of DMSO is added to each well and incubated for 2 h at room temperature (on a rocking platform, dark) to dissolve the formazan crystals. Absorbance was measured at 570 nm on a Synergy™ HTX Multi-Mode microplate Reader. All test concentrations were performed in 3 replicates. The percent viable cells (calculated based on absorbance of the control untreated cells) were plotted as a function of concentration (logarithmic scale) to obtain the IC_50_ values. Each experiment was repeated at least thrice (with 3 parallel replicates each time).

## 4. Conclusions

A novel series of thiazolidines have been prepared via a convenient and efficient method using surface-mediated acceleration and their anti-proliferative activity evaluated. The substitution of methylene at position 4 in thiazolidine ring blocks the aerobic oxidation to the corresponding thiazolidinone but still resulted in compounds unstable at room temperature. All the compounds were analyzed through spectroscopic analysis and the exact configuration of the thiazolidines was confirmed by X-ray single crystal characterization for one compound **5f** as an example for the synthesized library. Freshly prepared thiazolidines were screened for their cytotoxicity against breast cancer cell line (MCF7) using an MTT assay. Most of the synthesized library show potent cytotoxicity against a breast cancer cell line (MCF7). (*Z*)-*N*-(4-chlorophenyl)-5-((*Z*)-4-methoxybenzylidene)-3,4-dimethylthiazolidin-2-imine (**5c**) is the best analogues exhibiting a 15-fold higher potency compared to cisplatin. A study of structure activity relationship shows that electron withdrawing groups attached to the exocyclic 2-arylamine of thiazolidine exhibit the greatest activity among two synthesized libraries thus far in the present work and previously published study in 2019. A stability study was performed for the compounds at room temperature, 4 °C and –10 °C, which illustrated the thiazolidines bearing an electron withdrawing groups show a good chemical stability and biological activity. Thus, the present report provides a promising approach toward the development of a new variety of thiazolidine scaffolds in terms of green/clean chemistry and biologically active molecules.

## Figures and Tables

**Table 1:** The synthesized thiazolidines **5a–l** and their IC_50_ against an MCF7 cell line.

	Compound	Ar	Yield (%)	IC_50_ (µM ± SMD)
1	**5a**	C_6_H_5_	60	0.50 ± 0.21
2	**5b**	4-BrC_6_H_4_	61	0.62 ± 0.24
3	**5c**	4-ClC_6_H_4_	61	0.27 ± 0.14
4	**5d**	4-FC_6_H_4_	45	0.50 ± 0.23
5	**5e**	4-NO_2_C_6_H_4_	95	1.15 ± 0.18
6	**5f**	4-CF_3_C_6_H_4_	58	16.32 ± 1.24
7	**5g**	4-MeC_6_H_4_	20	ND †
8	**5h**	4-Me-3-ClC_6_H_3_	46	1.82 ± 0.17
9	**5i**	4-OMeC_6_H_4_	63	1.75 ± 0.16
10	**5j**	4-^t^BuC_6_H_4_	95	12.61 ± 3.71
11	**5k**	4-OMe-2-NO_2_C_6_H_3_	63	1.38 ± 0.13
12	**5l**	4-Br-2-CF_3_C_6_H_3_	57	15.64 ± 3.6
13	Cisplatin	-	-	4.14 ± 1.14

† This compound was not stable, and thus, the cytotoxicity was not determined.

## Data Availability

Not applicable.

## Supplementary Materials

The following supporting information can be downloaded online. Table S1. Crystal data and structure refinement.
