# Supplementary material for: One-Pot Synthesis of Novel 2-Imino-5-Arylidine-Thiazolidine Analogues and Evaluation of Their Anti-Proliferative Activity against MCF7 Breast Cancer Cell Line
Source: Molecules. 2022 Jan 27;27(3):841. doi: 10.3390/molecules27030841 (PMC8840064; doi:10.3390/molecules27030841)
Supplement: Supplementary file 1 [file molecules-27-00841-s001.zip › molecules-1487979-supplementary.pdf]

One-pot synthesis of novel 2-imino-5-arylidine-thiazolidine analogues and evaluation of their anti-proliferative activity against MCF7 breast cancer cell line

Marian N. Aziz, Arzoo Patel, Amany Iskander, Avisankar Chini, Delphine Gout, Subhrangsu S. Mandal, Carl J. Lovely

Department of Chemistry and Biochemistry, University of Texas Arlington, Arlington, TX 76019

Supporting Information

1- X-ray study of thiazolidine 5f:

The single crystal X-ray diffraction studies were carried out on a Bruker Kappa APEX-II CCD diffractometer at 100(2) K using monochromated Mo-Kα radiation (λ = 0.71073 Å) and a detector-to-crystal distance of 5.220 cm. A 0.17 x 0.045 x 0.030 mm colorless needle was mounted on a Cryoloop with Nujol oil. Data were collected in a hemisphere or full sphere of reciprocal space with 0.3° scans in ω for an exposure time of 30 s per frame up to a maximum 2θ value of 56.66°. A total of 3759 reflections were collected covering the indices, $-13 \leq h \leq 13$, $-29 \leq k \leq 28$, $-12 \leq l \leq 12$. 2336 reflections were found to be symmetry independent. Indexing and unit cell refinement indicated a monoclinic lattice. The space group was found to be $P2_1/c$. The measured intensities were corrected for Lorenz and polarization effects and were further corrected for absorption using the multi-scan method SCALE3 ABSPACK. Based on the data, structural model was obtained by direct method using the Superflip subroutine implemented in the JANA2006 software package. The refinement was performed via full-matrix least-squares on F^2 by using the JANA2006 software package. Crystallographic data are summarized in Table 1.

Table S1. Crystal data and structure refinement.

Identification code		
Empirical formula	$C_{20}H_{20}O_1F_3N_2S_1$	
Formula weight	392.4 g/mol	
Temperature	100(2) K	
Wavelength	0.71073 Å	
Crystal system	Monoclinic	
Space group	$P2_1/c$	
Unit cell dimensions	a = 10.0749(11) Å	α= 90 °
	b = 21.9542(13) Å	β= 112.694(3) °
	c = 9.3291(11) Å	γ = 90 °
Volume	1903.7 (7) $Å^3$	
Z	4	
Density (calculated)	1.3692 g/cm^3	
Absorption coefficient	2.10 mm^{-1}	
F(000)	816	
Crystal size	0.170 x 0.045 x 0.030 mm^3	
Crystal color, habit	Colorless needle	

Theta range for data collection	2.19 to 28.33°
Index ranges	-13≤h≤13, -29≤k ≤28, -12≤l≤12
Reflections collected	3759
Independent reflections	2336 [R(int) = 15.44 %, R(sigma) = 13.93%]
Completeness to theta = 28.13°	100 %
Absorption correction	multi-scan
Max. and min. transmission	0.978 and 0.994
Refinement method	Full-matrix least-squares on F^2
Data / restraints / parameters	2336/0/76
Goodness-of-fit on F^2	1.38
Final R indices [I>3sigma(I)]	R1 = 5.37, wR2 = 3.98
R indices (all data)	R1 = 5.37, wR2 = 3.98
Extinction coefficient	n/a
Largest diff. peak and hole	-0.06 and + 0.07 $e^- Å^{-3}$

3- Copies of ^{1}H and ^{13}C NMR spectra:

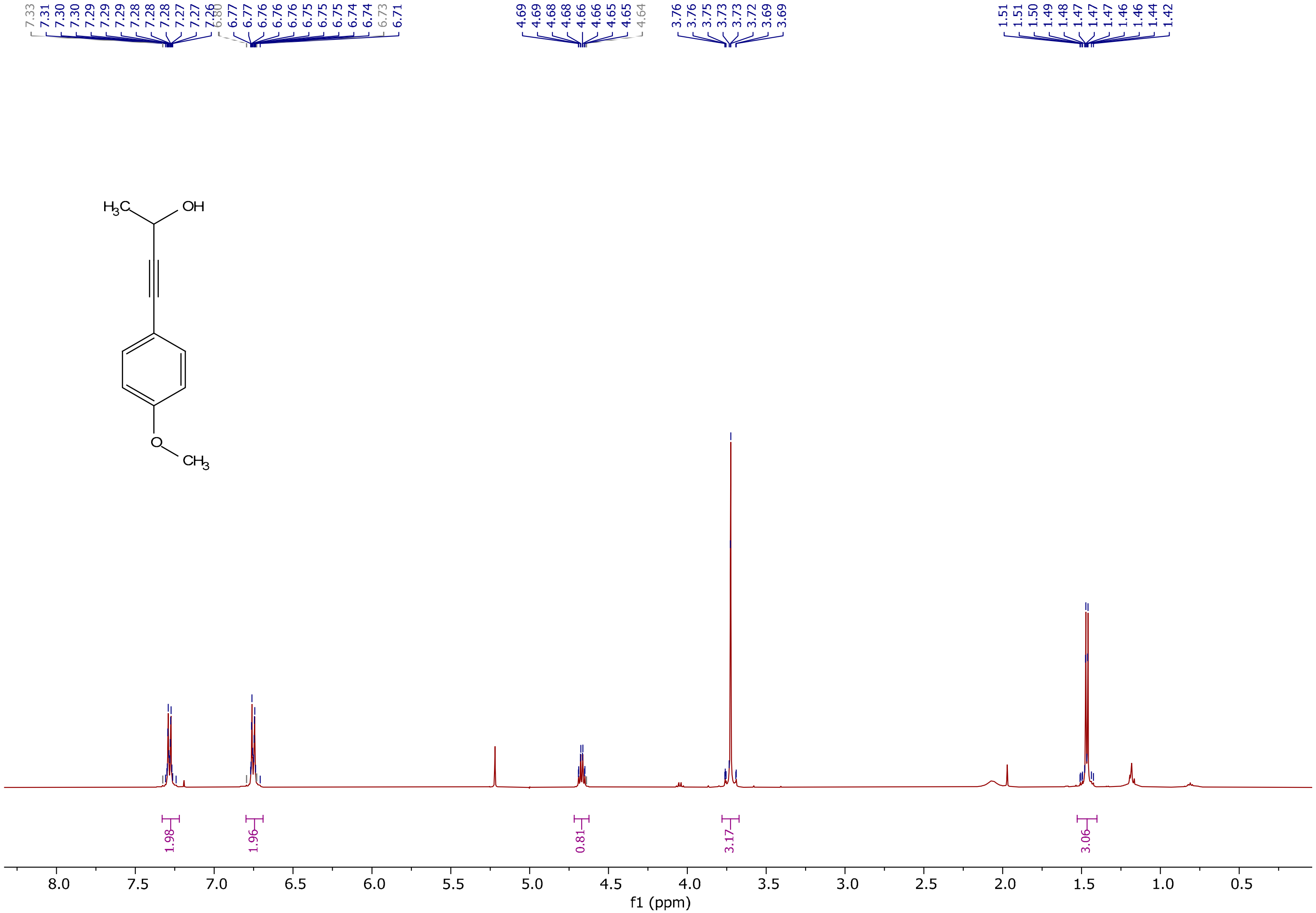

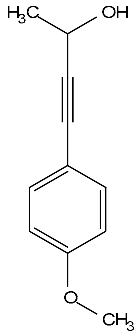

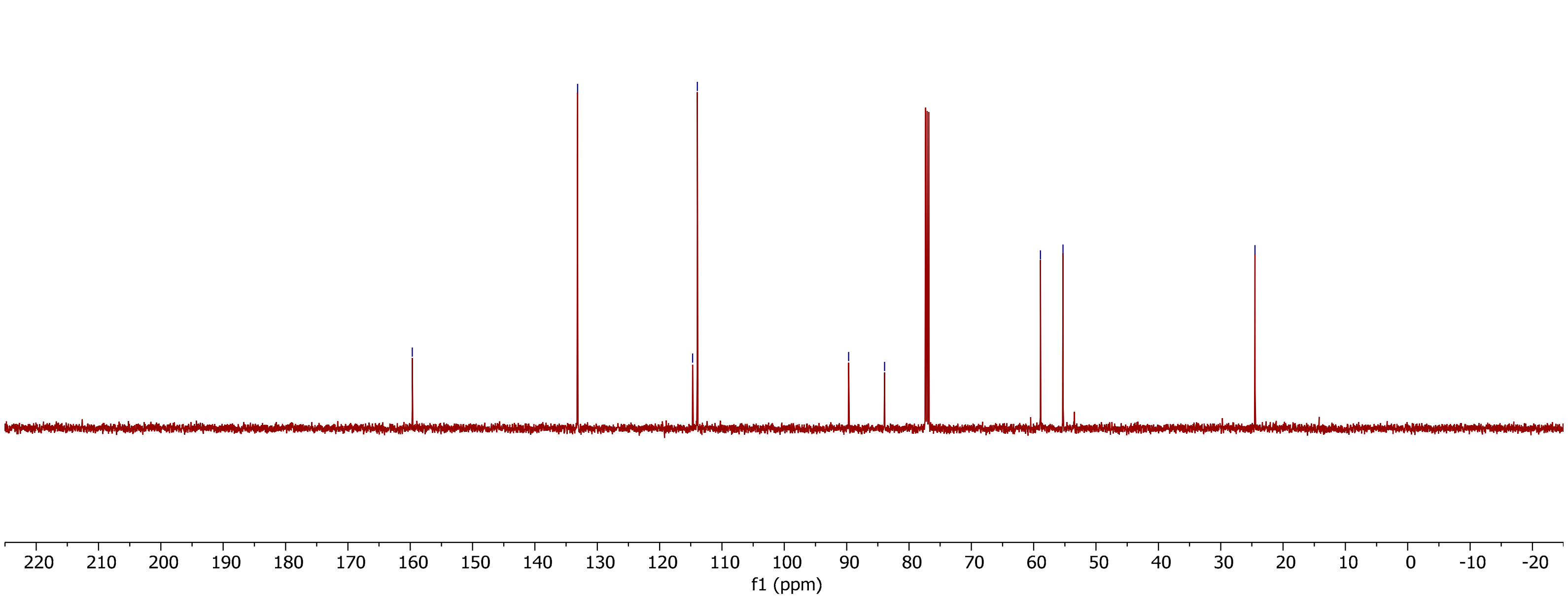
159.67
133.16
114.71
113.94
89.67
83.92
58.91
55.30
24.50
220
210
200
190
180
170
160
150
140
130
120
110
100
90
80
70
60
50
40
30
20
10
0
-10
-20
f1 (ppm)

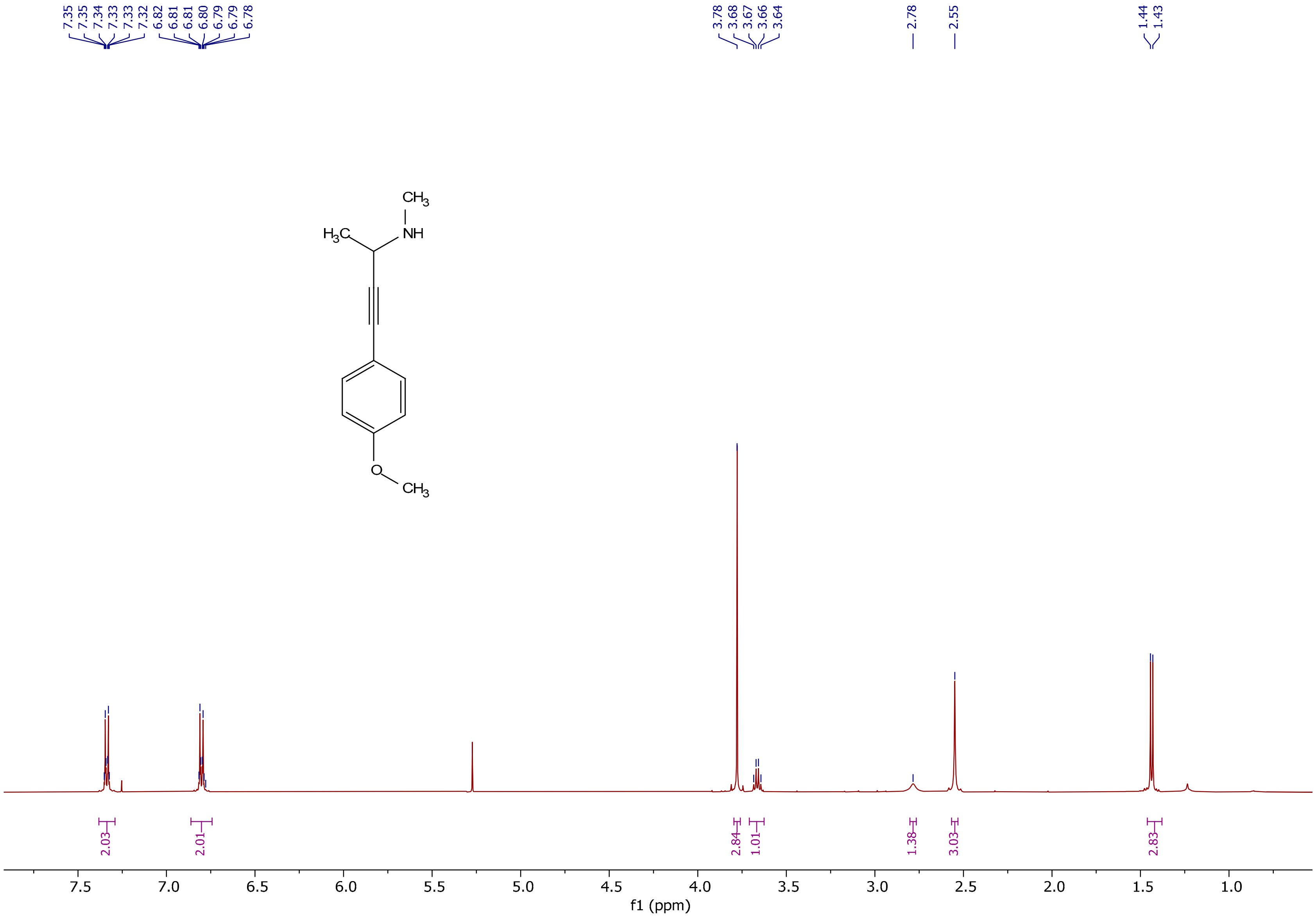
7.35
7.35
7.34
7.33
7.33
7.32
6.82
6.81
6.81
6.80
6.79
6.79
6.78
3.78
3.68
3.67
3.66
3.64
2.78
2.55
1.44
1.43
CH3
NH
H3C
O
CH3
2.03
2.01
2.84
1.01
1.38
3.03
2.83
7.5
7.0
6.5
6.0
5.5
5.0
4.5
4.0
3.5
3.0
2.5
2.0
1.5
1.0
f1 (ppm)

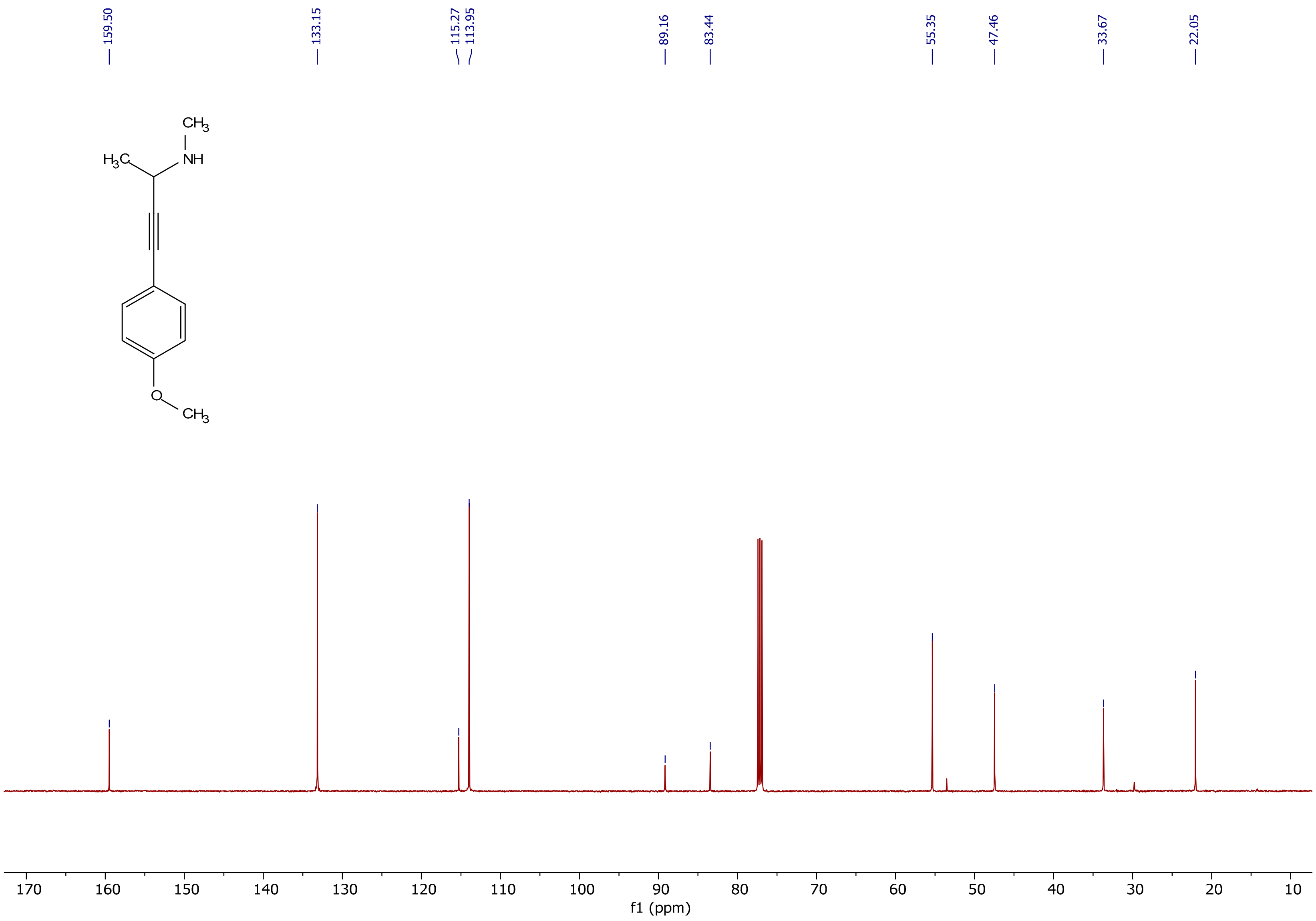
159.50
133.15
115.27
113.95
89.16
83.44
55.35
47.46
33.67
22.05
CH3
H3C
NH
O
CH3
170
160
150
140
130
120
110
100
90
80
70
60
50
40
30
20
10
f1 (ppm)

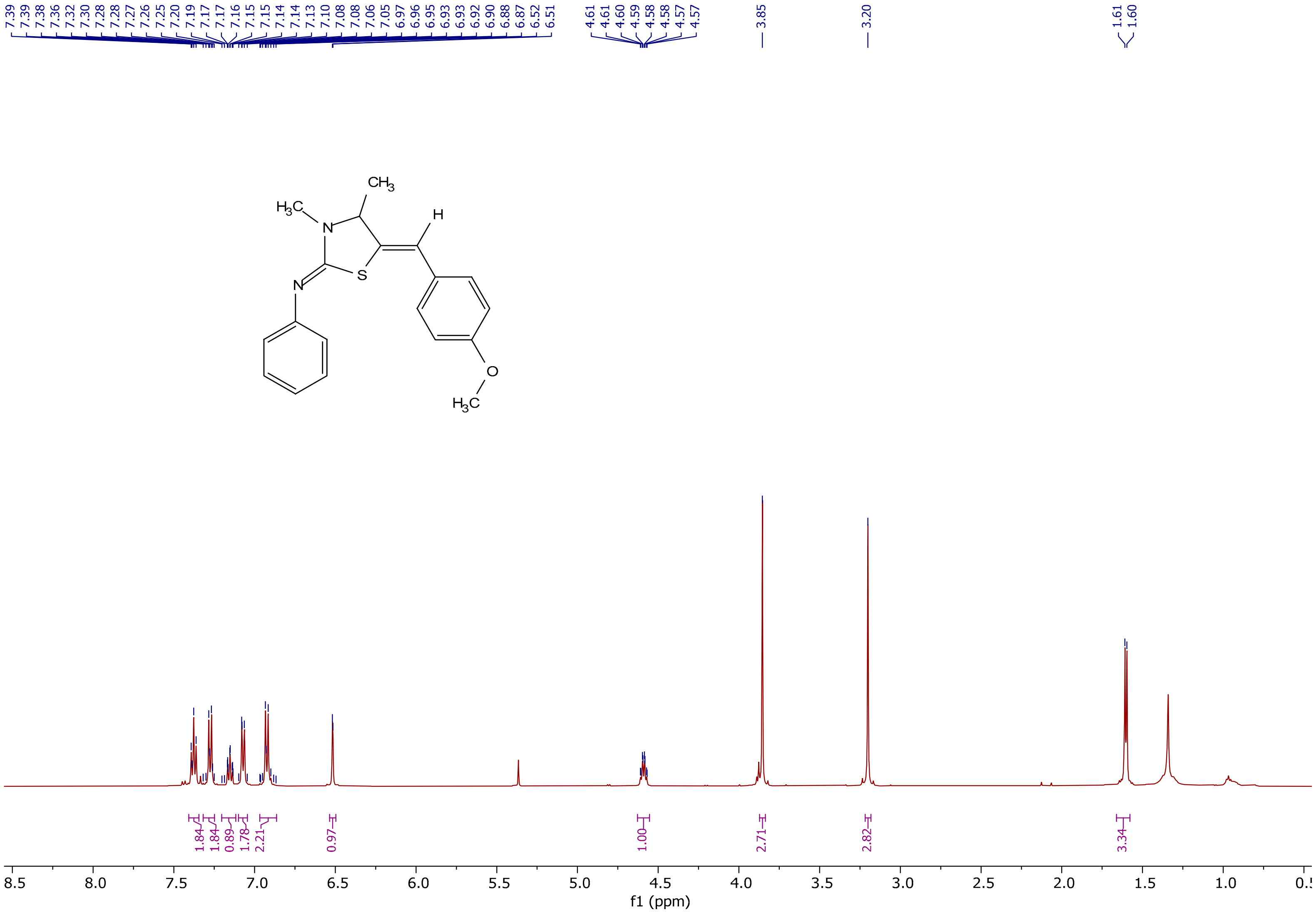
7.39 7.39 7.38 7.36 7.32 7.30 7.28 7.28 7.27 7.26 7.25 7.20 7.19 7.17 7.16 7.15 7.14 7.14 7.13 7.10 7.08 7.08 7.06 7.05 6.97 6.96 6.95 6.93 6.93 6.92 6.90 6.88 6.87 6.52 6.51
4.61 4.61 4.60 4.59 4.58 4.58 4.57 4.57
3.85
3.20
1.61 1.60
CH3
H3C
N
H
S
N
O
H3C
1.84 1.84 0.89 1.78 2.21
0.97
1.00
2.71
2.82
3.34
8.5 8.0 7.5 7.0 6.5 6.0 5.5 5.0 4.5 4.0 3.5 3.0 2.5 2.0 1.5 1.0 0.5
f1 (ppm)

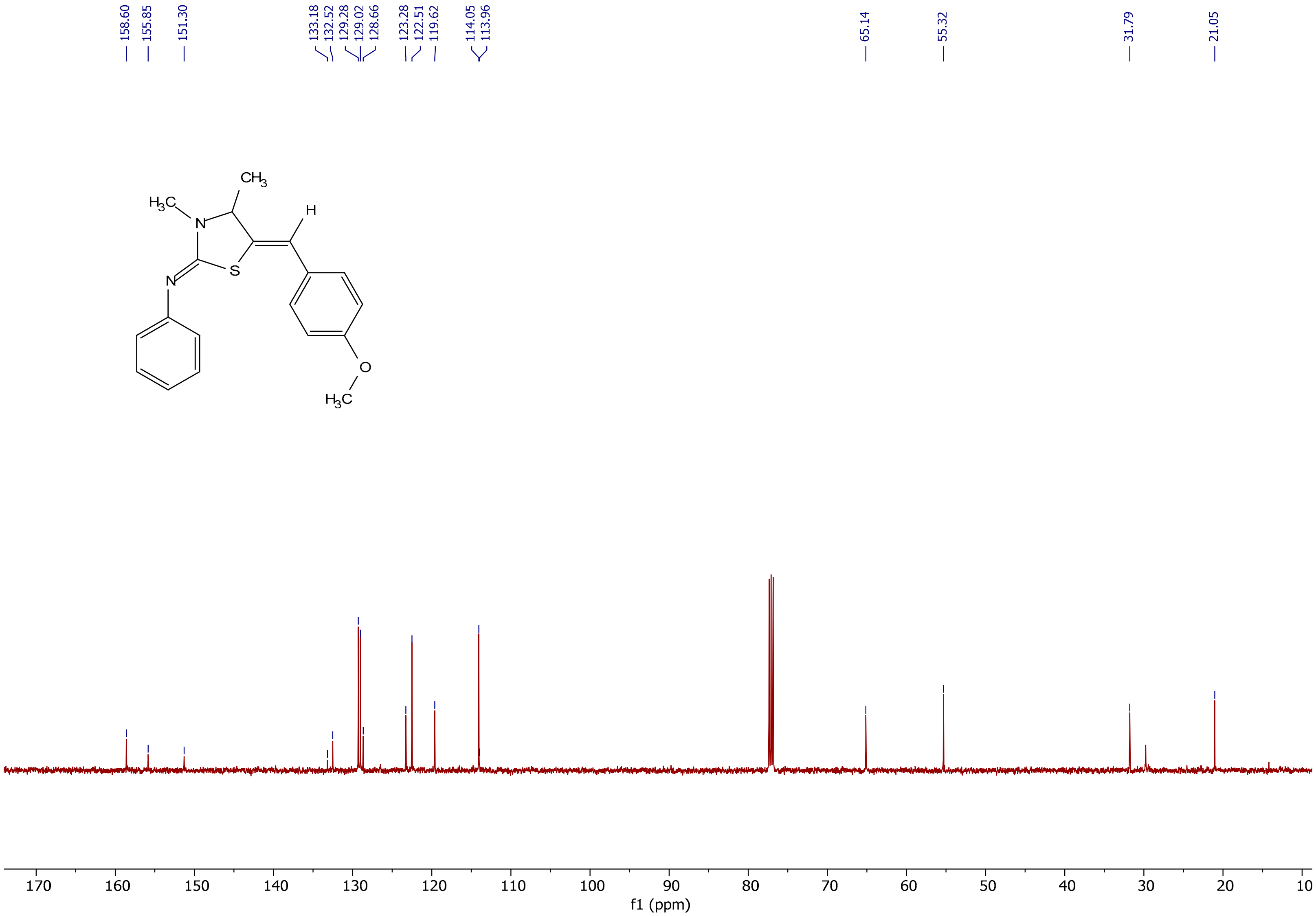
158.60
155.85
151.30
133.18
132.52
129.28
129.02
128.66
123.28
122.51
119.62
114.05
113.96
65.14
55.32
31.79
21.05
CH3
H3C
N
H
S
N
O
H3C
170
160
150
140
130
120
110
100
90
80
70
60
50
40
30
20
10
f1 (ppm)

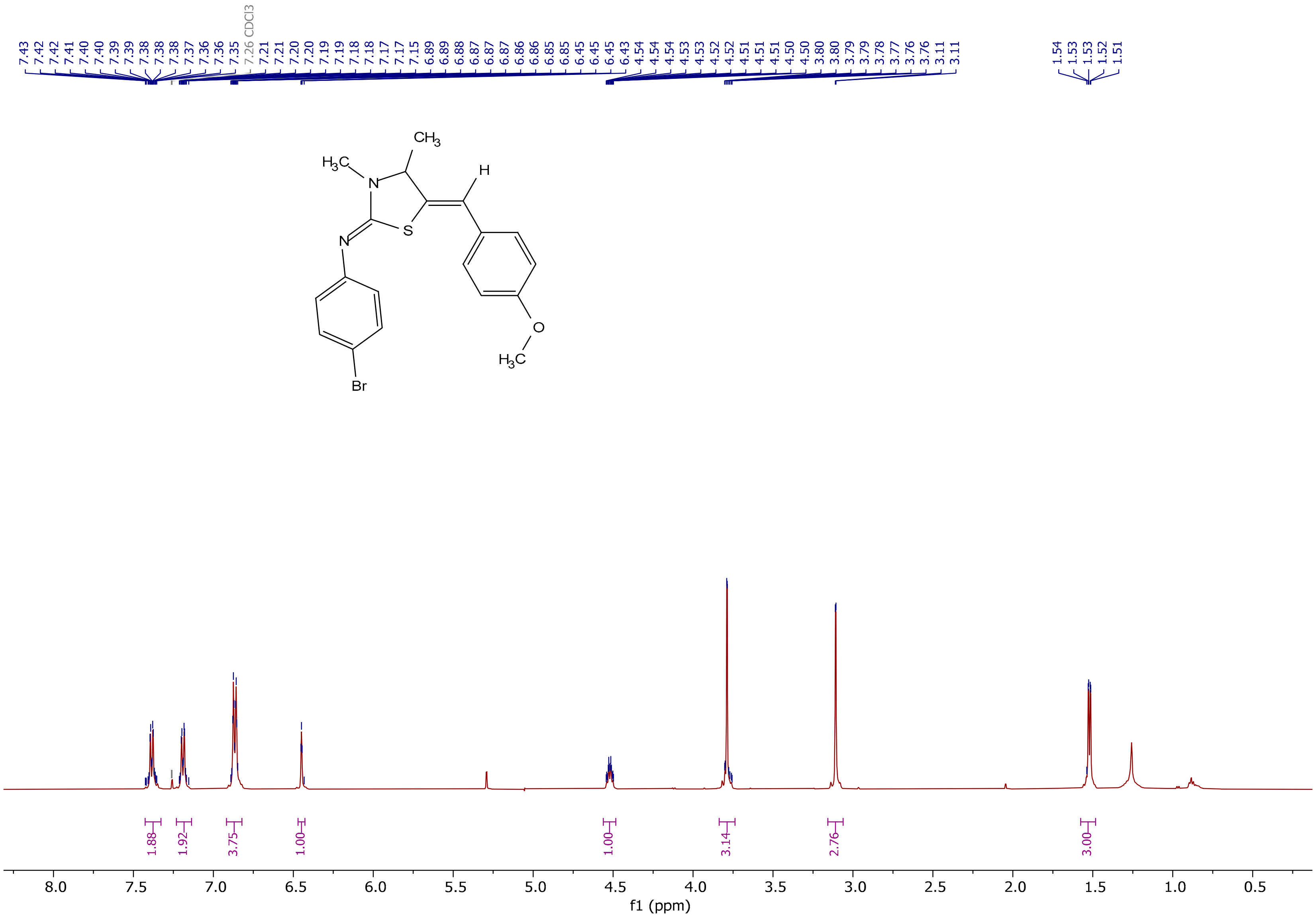
CH3
H3C
N
H
S
N
O
H3C
Br
7.43 7.42 7.42 7.41 7.40 7.40 7.39 7.39 7.38 7.38 7.38 7.37 7.36 7.36 7.35 7.26 CDCl3 7.21 7.21 7.20 7.20 7.19 7.19 7.18 7.18 7.17 7.17 7.15 6.89 6.89 6.88 6.87 6.87 6.87 6.86 6.86 6.85 6.85 6.45 6.45 6.45 6.43 4.54 4.54 4.54 4.53 4.53 4.52 4.52 4.51 4.51 4.51 4.50 4.50 3.80 3.80 3.79 3.79 3.78 3.77 3.76 3.76 3.11 3.11 1.54 1.53 1.53 1.52 1.51
1.88 1.92 3.75 1.00 1.00 3.14 2.76 3.00
8.0 7.5 7.0 6.5 6.0 5.5 5.0 4.5 4.0 3.5 3.0 2.5 2.0 1.5 1.0 0.5
f1 (ppm)

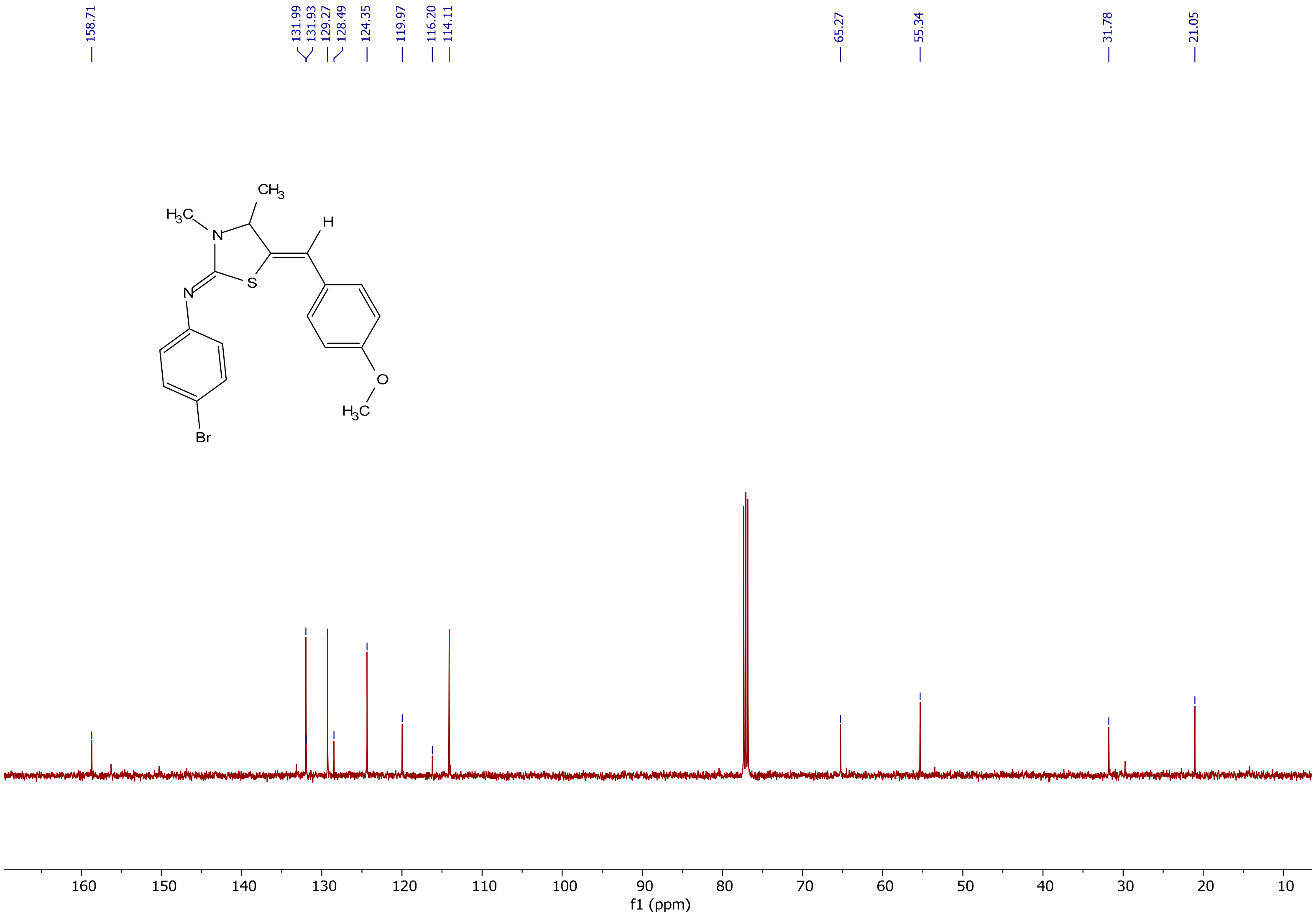
158.71
131.99
131.93
129.27
128.49
124.35
119.97
116.20
114.11
65.27
55.34
31.78
21.05
CH3
H3C
N
H
S
N
O
H3C
Br
160
150
140
130
120
110
100
90
80
70
60
50
40
30
20
10
f1 (ppm)

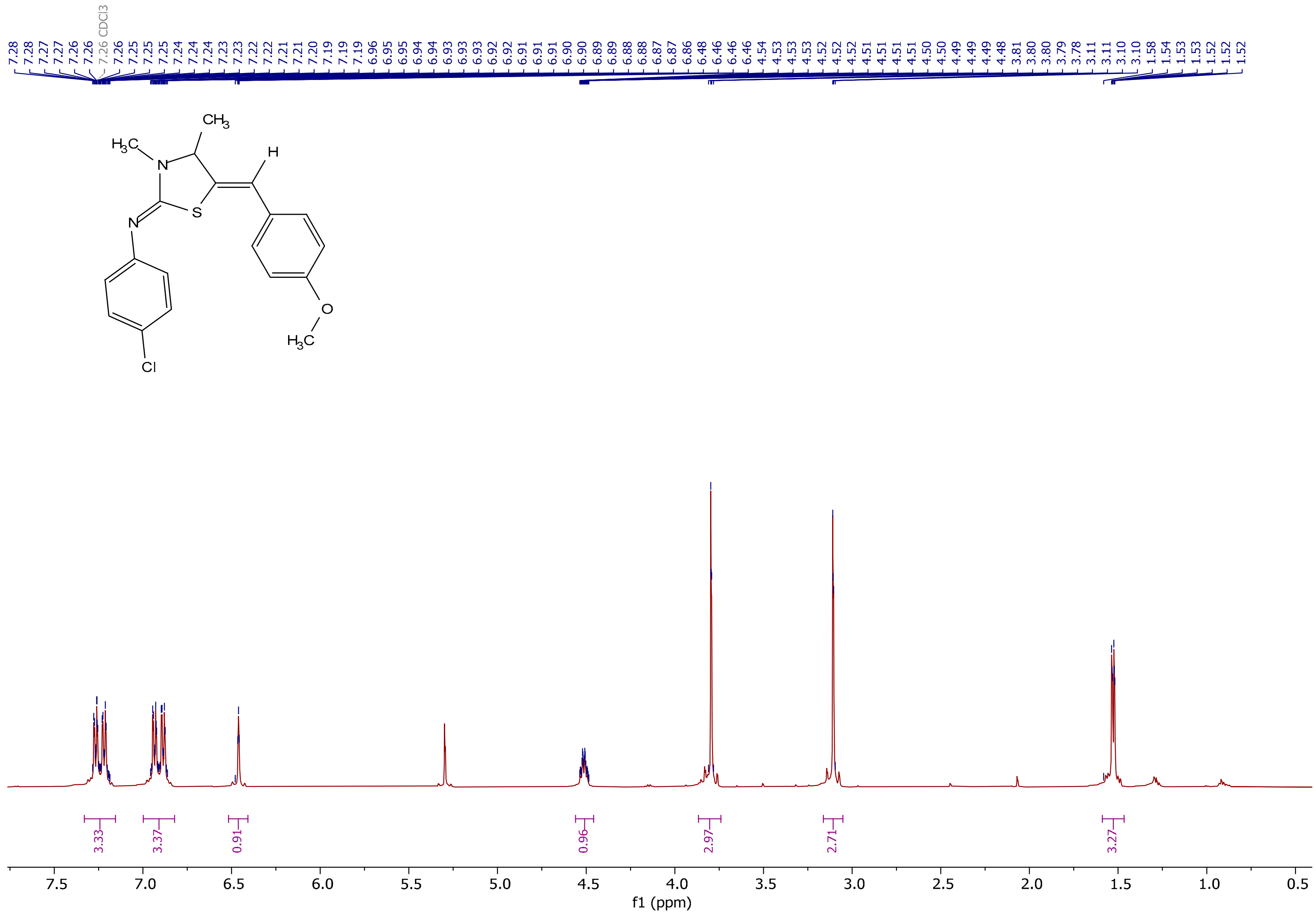
7.28 7.28 7.27 7.27 7.26 7.26 7.26 CDCl3 7.26 7.25 7.25 7.25 7.24 7.24 7.24 7.23 7.23 7.22 7.22 7.21 7.21 7.20 7.19 7.19 6.96 6.95 6.95 6.94 6.94 6.93 6.93 6.93 6.92 6.92 6.91 6.91 6.91 6.90 6.89 6.89 6.88 6.88 6.87 6.87 6.86 6.48 6.46 6.46 6.46 4.54 4.53 4.53 4.53 4.52 4.52 4.52 4.51 4.51 4.51 4.51 4.50 4.50 4.49 4.49 4.48 3.81 3.80 3.80 3.79 3.78 3.11 3.11 3.10 3.10 1.58 1.54 1.53 1.53 1.52 1.52 1.52
CH3
H3C
N
H
S
N
Cl
O
H3C
3.33
3.37
0.91
0.96
2.97
2.71
3.27
7.5 7.0 6.5 6.0 5.5 5.0 4.5 4.0 3.5 3.0 2.5 2.0 1.5 1.0 0.5
f1 (ppm)

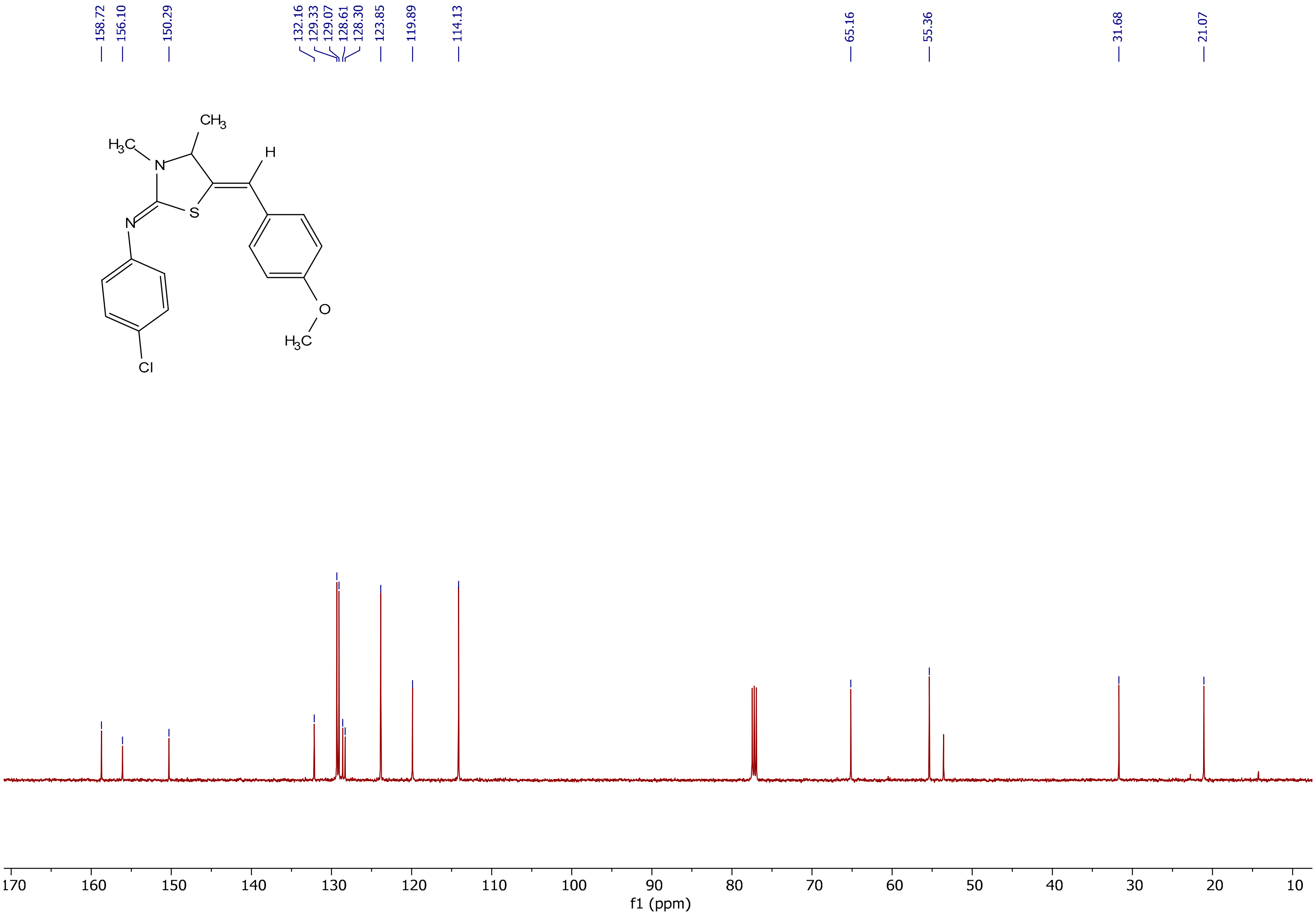

158.72
156.10
150.29
132.16
129.33
129.07
128.61
128.30
123.85
119.89
114.13
65.16
55.36
31.68
21.07
CH3
H3C
N
H
S
N
O
H3C
Cl
170 160 150 140 130 120 110 100 90 80 70 60 50 40 30 20 10
f1 (ppm)

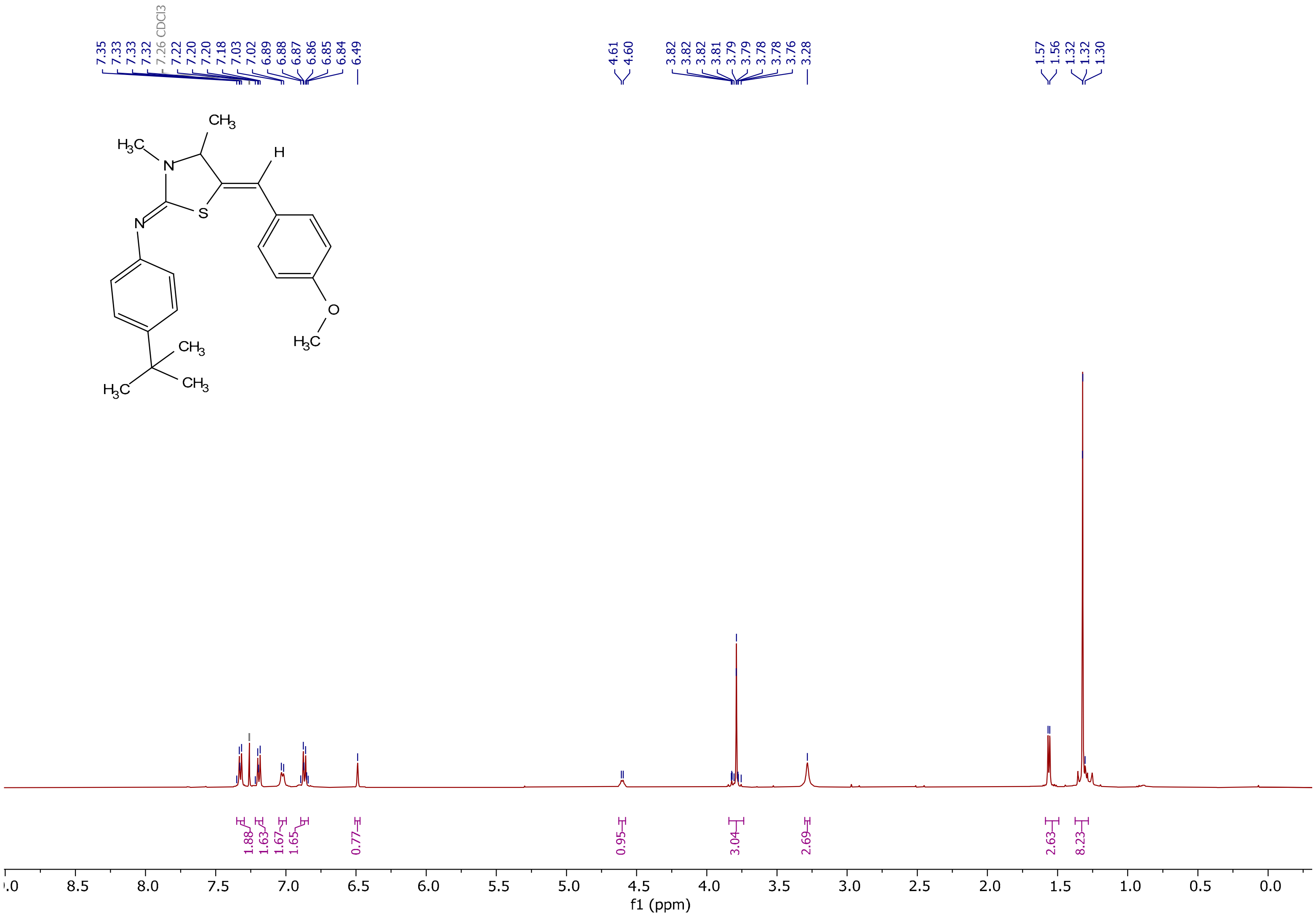
7.35
7.33
7.33
7.32
7.26 CDCl3
7.22
7.20
7.20
7.18
7.03
7.02
6.89
6.88
6.87
6.86
6.85
6.84
6.49
4.61
4.60
3.82
3.82
3.82
3.81
3.79
3.79
3.78
3.78
3.76
3.28
1.57
1.56
1.32
1.32
1.30
CH3
H3C
N
H
S
N
O
H3C
CH3
H3C
CH3
1.88
1.63
1.67
1.65
0.77
0.95
3.04
2.69
2.63
8.23
8.5
8.0
7.5
7.0
6.5
6.0
5.5
5.0
4.5
4.0
3.5
3.0
2.5
2.0
1.5
1.0
0.5
0.0
f1 (ppm)

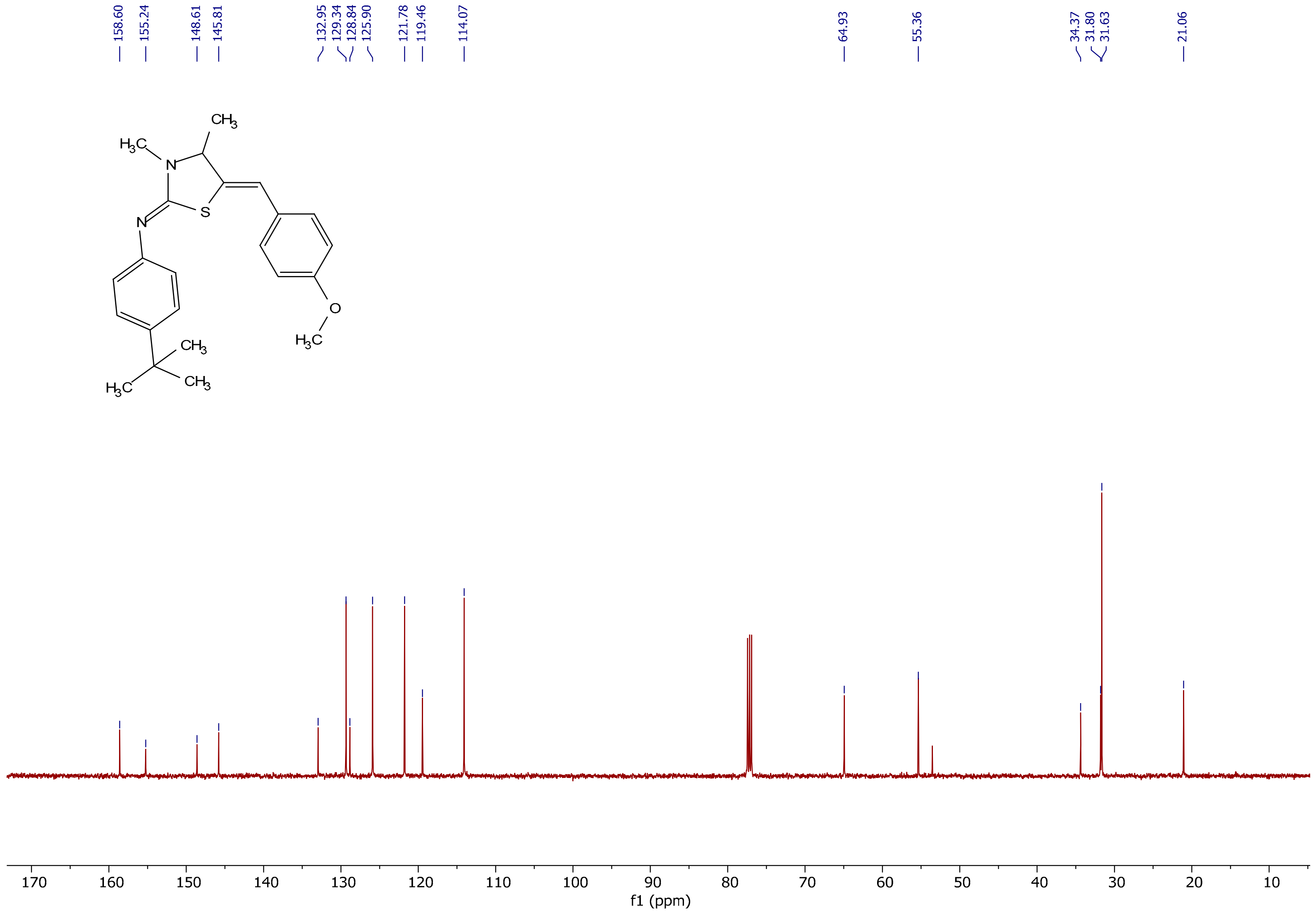
158.60
155.24
148.61
145.81
132.95
129.34
128.84
125.90
121.78
119.46
114.07
64.93
55.36
34.37
31.80
31.63
21.06
f1 (ppm)

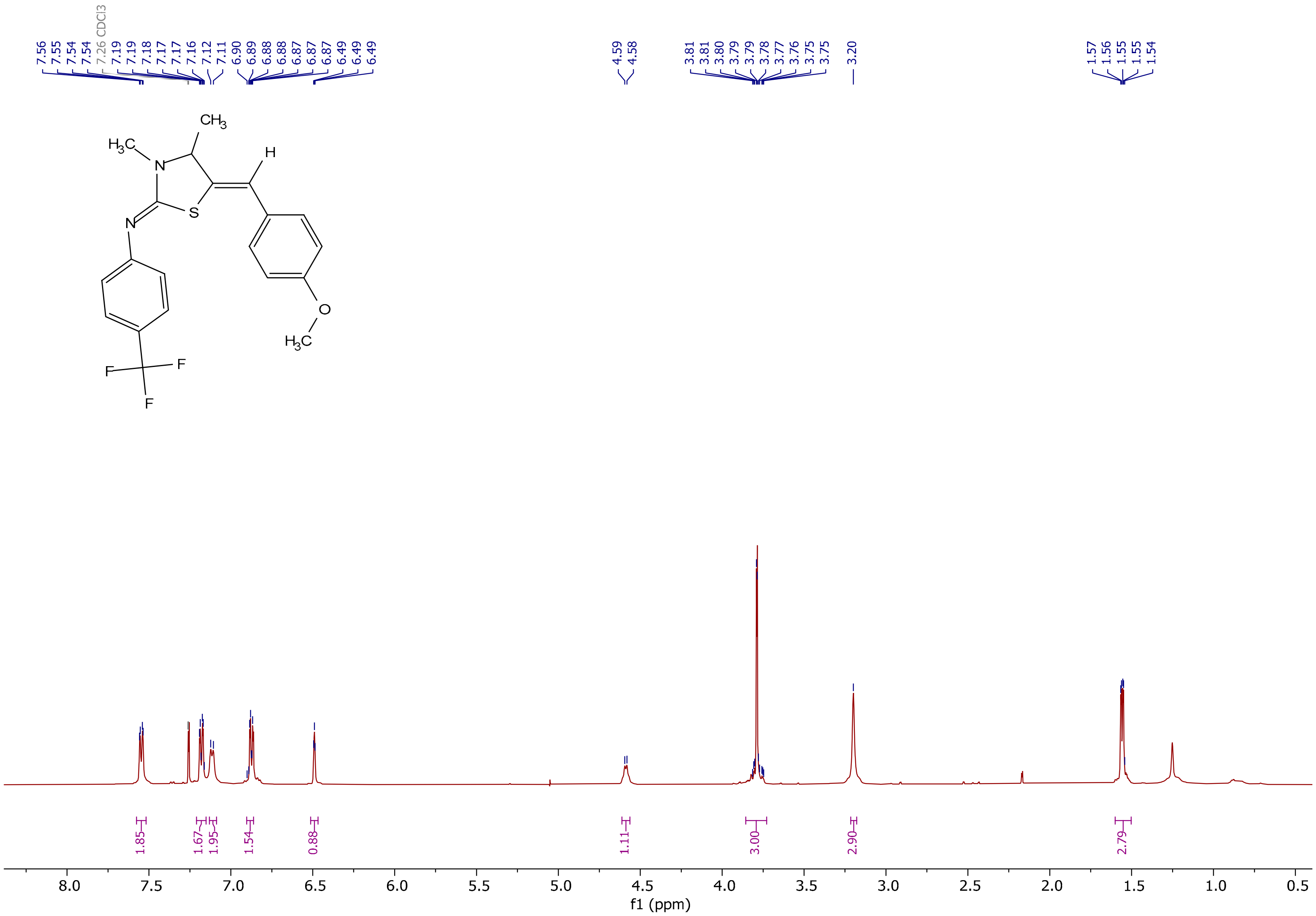

7.56
7.55
7.54
7.54
7.26 CDCl3
7.19
7.19
7.18
7.17
7.17
7.16
7.12
7.11
6.90
6.89
6.88
6.88
6.87
6.87
6.87
6.49
6.49
6.49
4.59
4.58
3.81
3.81
3.80
3.79
3.79
3.78
3.77
3.76
3.75
3.75
3.20
1.57
1.56
1.55
1.55
1.54
CH3
H3C
N
H
S
N
O
H3C
F
F
F
1.85
1.67
1.95
1.54
0.88
1.11
3.00
2.90
2.79
8.0
7.5
7.0
6.5
6.0
5.5
5.0
4.5
4.0
3.5
3.0
2.5
2.0
1.5
1.0
0.5
f1 (ppm)

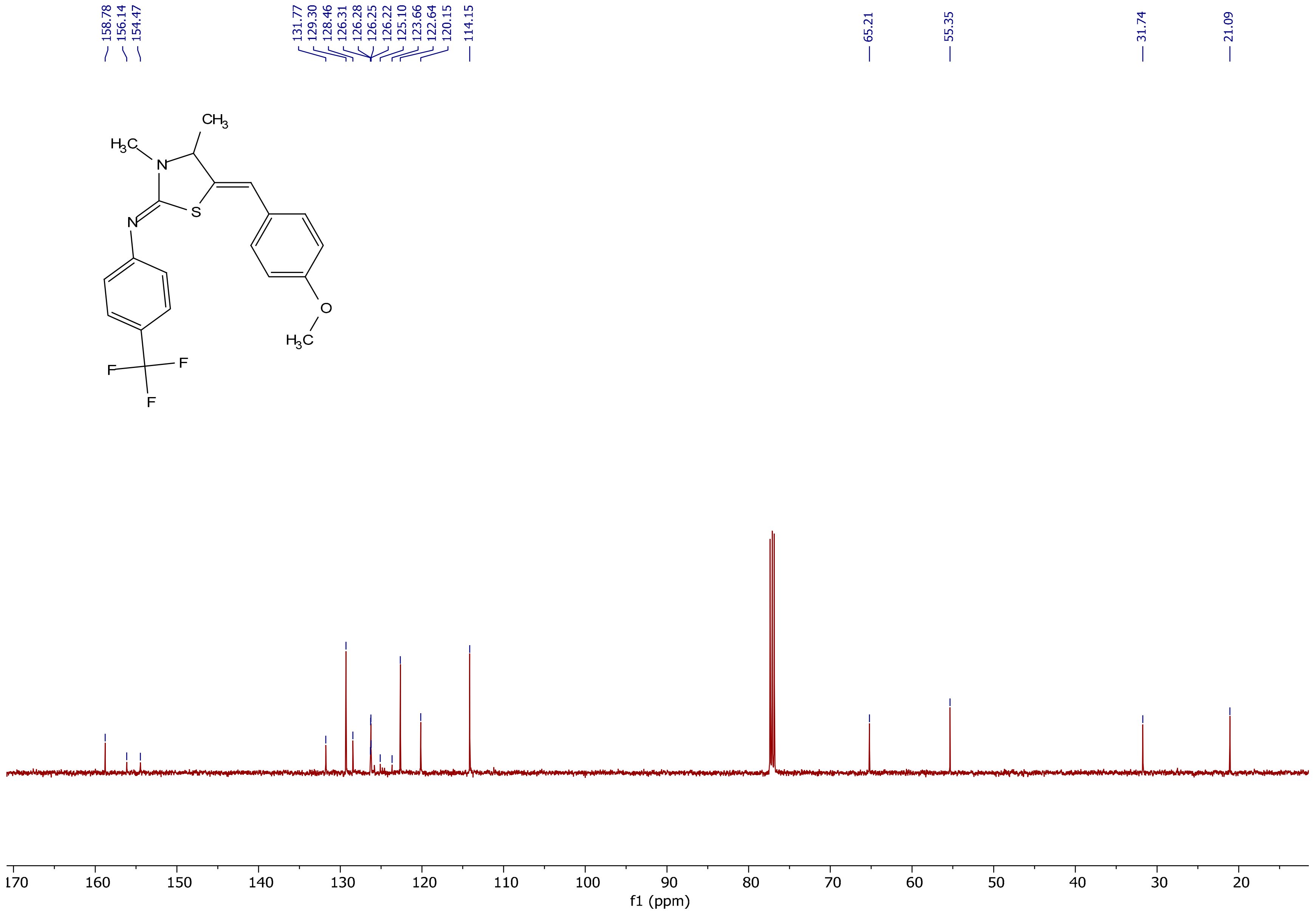

158.78
156.14
154.47
131.77
129.30
128.46
126.31
126.28
126.25
126.22
125.10
123.66
122.64
120.15
114.15
65.21
55.35
31.74
21.09
CH3
H3C
N
S
N
O
H3C
F
F
F
170
160
150
140
130
120
110
100
90
80
70
60
50
40
30
20
f1 (ppm)

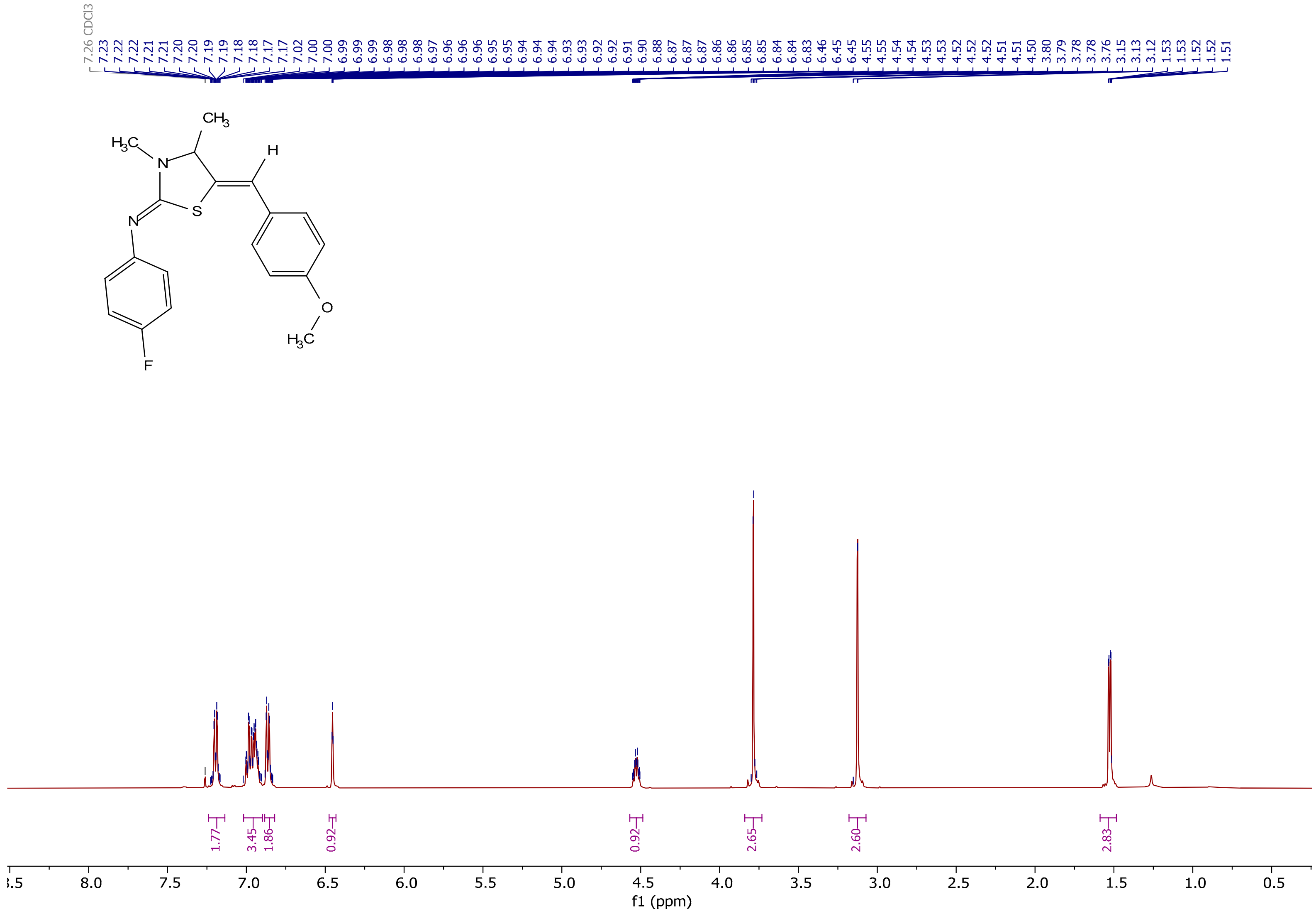
7.26 CDCl3
7.23 7.22 7.22 7.21 7.21 7.20 7.20 7.19 7.19 7.18 7.18 7.17 7.17 7.02 7.00 7.00 6.99 6.99 6.99 6.98 6.98 6.98 6.97 6.96 6.96 6.95 6.95 6.94 6.94 6.94 6.93 6.93 6.92 6.92 6.91 6.90 6.88 6.87 6.87 6.86 6.86 6.85 6.85 6.84 6.84 6.83 6.46 6.45 6.45 4.55 4.55 4.54 4.54 4.53 4.53 4.52 4.52 4.52 4.51 4.51 4.50 3.80 3.79 3.78 3.78 3.76 3.15 3.13 3.12 1.53 1.53 1.52 1.52 1.51
CH3
H3C
N
S
H
N
O
H3C
F
1.77
3.45
1.86
0.92
0.92
2.65
2.60
2.83
8.5 8.0 7.5 7.0 6.5 6.0 5.5 5.0 4.5 4.0 3.5 3.0 2.5 2.0 1.5 1.0 0.5
f1 (ppm)

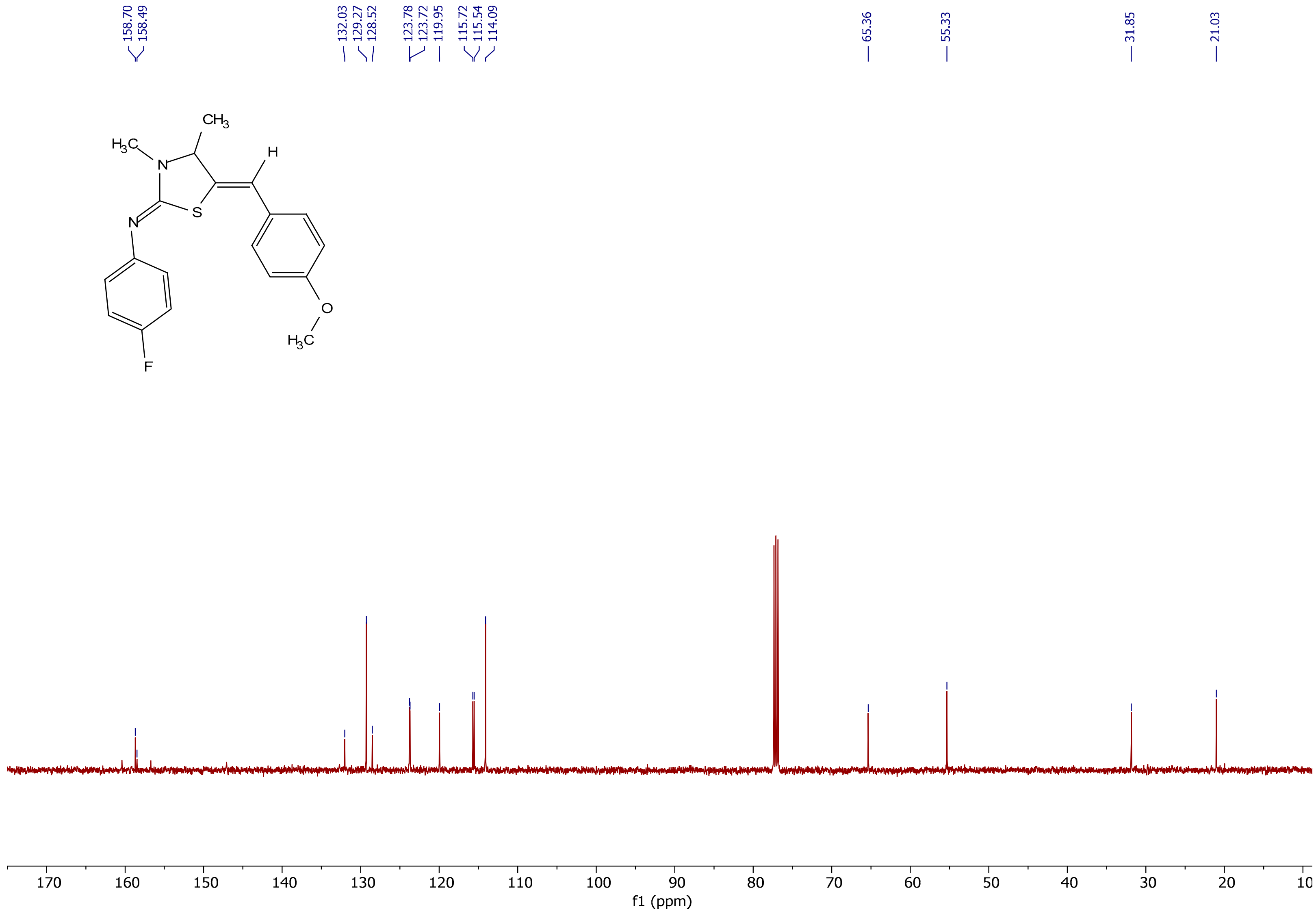
158.70
158.49
132.03
129.27
128.52
123.78
123.72
119.95
115.72
115.54
114.09
65.36
55.33
31.85
21.03
CH3
H3C
N
H
S
N
O
H3C
F
170
160
150
140
130
120
110
100
90
80
70
60
50
40
30
20
10
f1 (ppm)

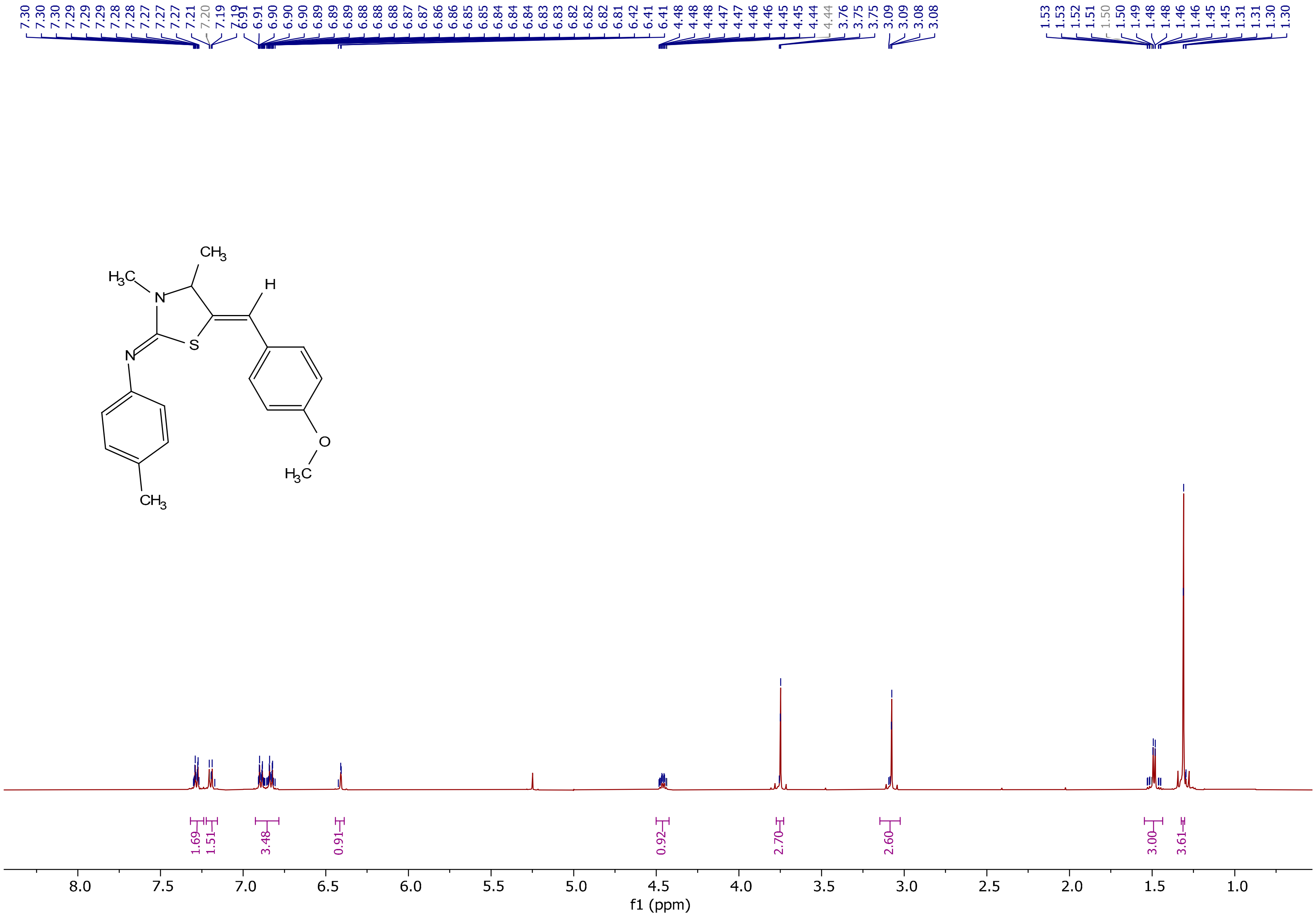
H3C
N
CH3
H
S
N
O
H3C
CH3
f1 (ppm)
1.69
1.51
3.48
0.91
0.92
2.70
2.60
3.00
3.61

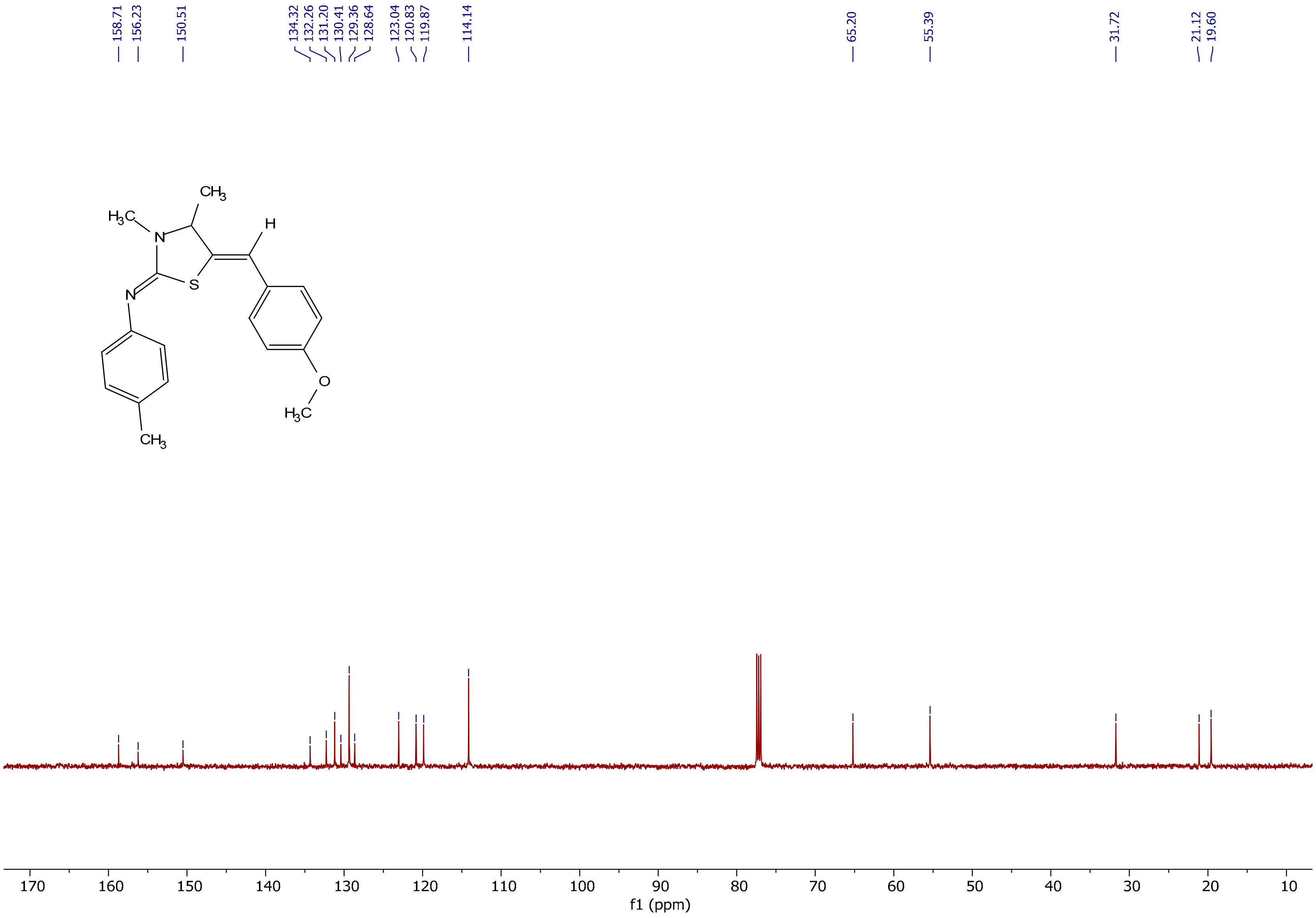
158.71
156.23
150.51
134.32
132.26
131.20
130.41
129.36
128.64
123.04
120.83
119.87
114.14
65.20
55.39
31.72
21.12
19.60
CH3
H3C
N
H
N
S
O
H3C
CH3
170 160 150 140 130 120 110 100 90 80 70 60 50 40 30 20 10
f1 (ppm)

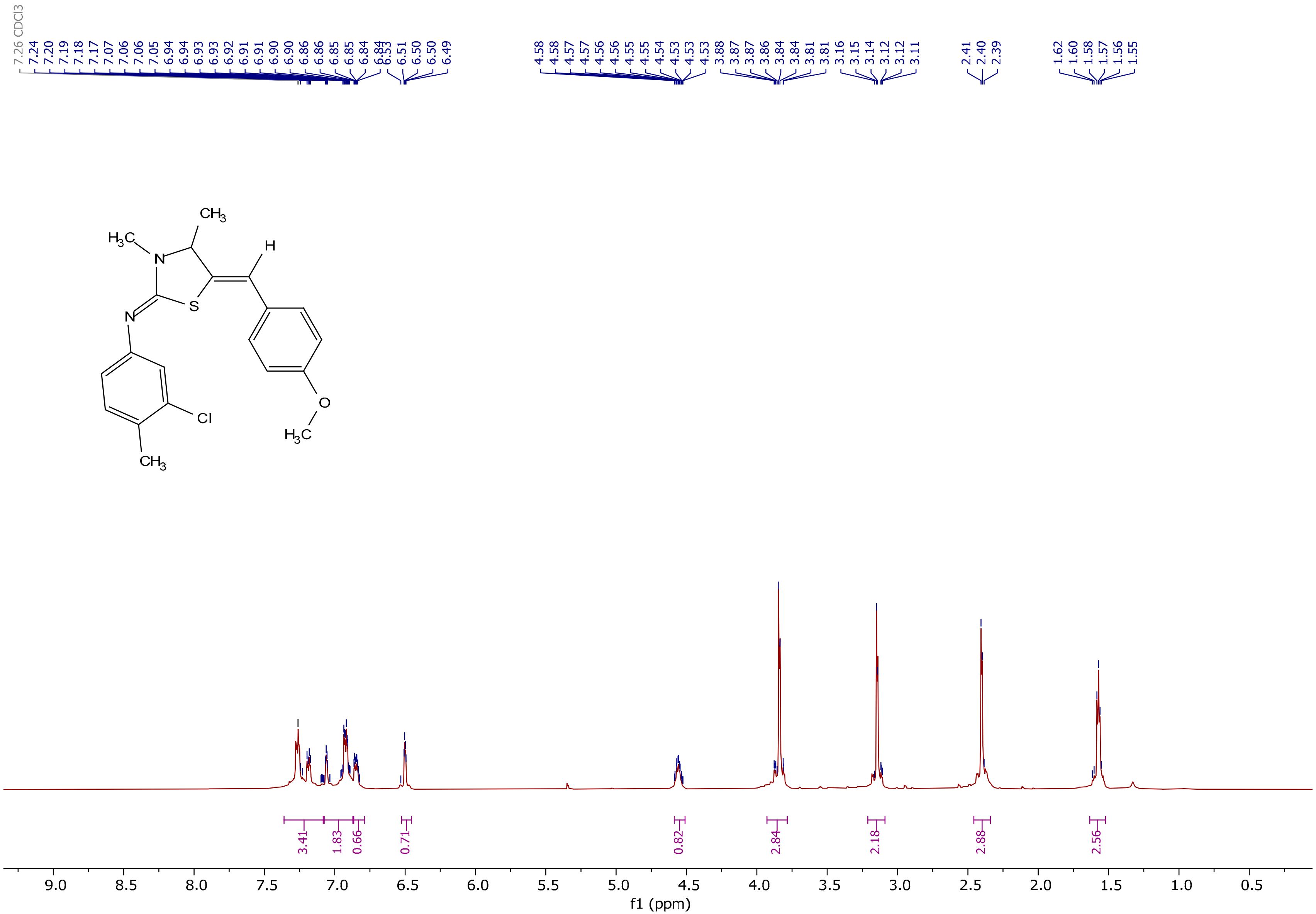
7.26 CDCl3
7.24 7.20 7.19 7.18 7.17 7.07 7.06 7.06 7.05 6.94 6.94 6.93 6.93 6.92 6.91 6.91 6.90 6.90 6.86 6.86 6.85 6.85 6.84 6.84 6.53 6.51 6.50 6.50 6.49
4.58 4.58 4.57 4.57 4.56 4.56 4.55 4.55 4.54 4.53 4.53 4.53 3.88 3.87 3.87 3.86 3.84 3.84 3.81 3.81 3.16 3.15 3.14 3.12 3.12 3.11
2.41 2.40 2.39
1.62 1.60 1.58 1.57 1.56 1.55
CH3
H3C
N
H
S
N
Cl
CH3
O
H3C
3.41
1.83
0.66
0.71
0.82
2.84
2.18
2.88
2.56
9.0 8.5 8.0 7.5 7.0 6.5 6.0 5.5 5.0 4.5 4.0 3.5 3.0 2.5 2.0 1.5 1.0 0.5
f1 (ppm)

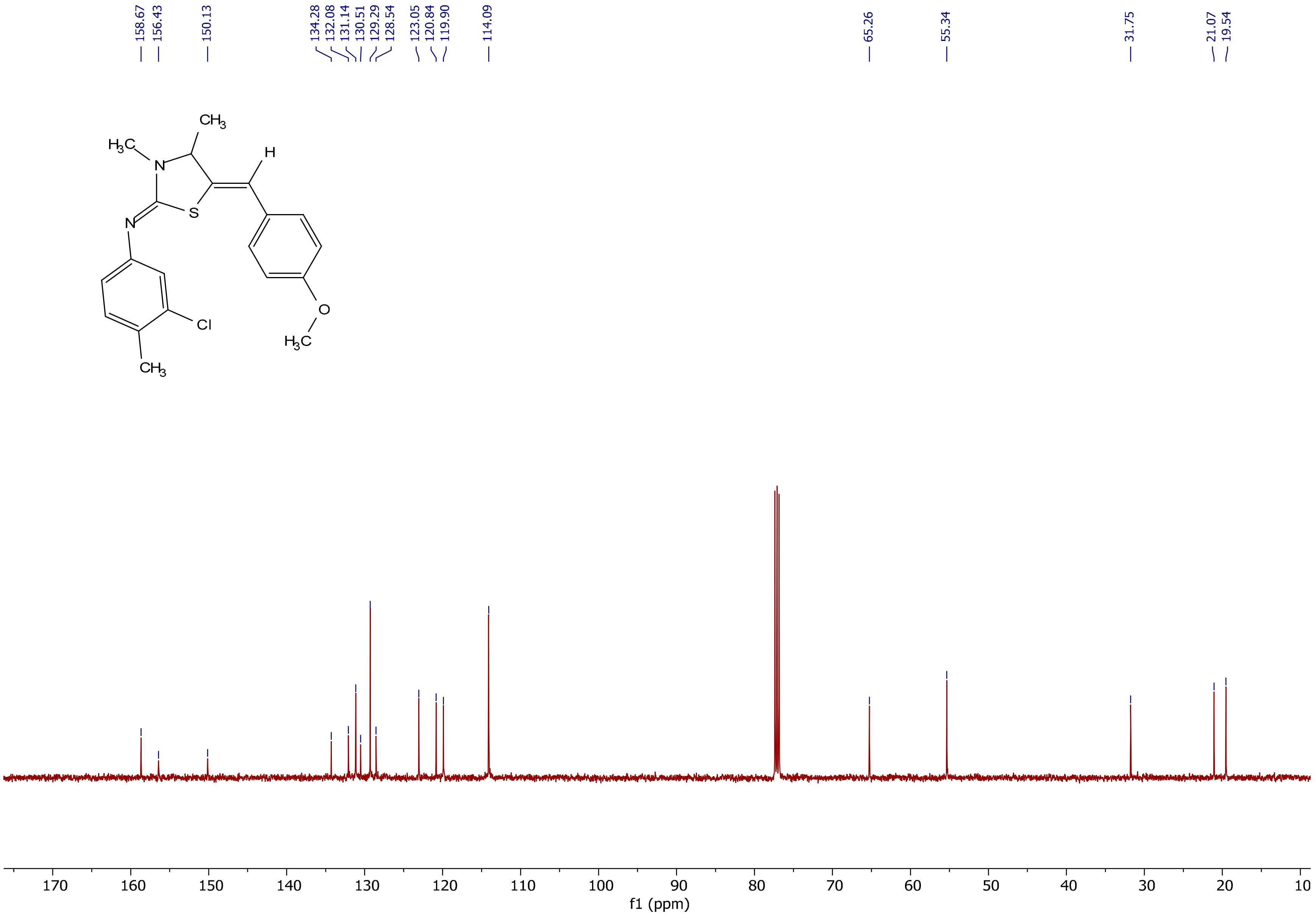

158.67
156.43
150.13
134.28
132.08
131.14
130.51
129.29
128.54
123.05
120.84
119.90
114.09
65.26
55.34
31.75
21.07
19.54
CH3
H3C
N
H
S
N
Cl
CH3
O
H3C
170
160
150
140
130
120
110
100
90
80
70
60
50
40
30
20
10
f1 (ppm)

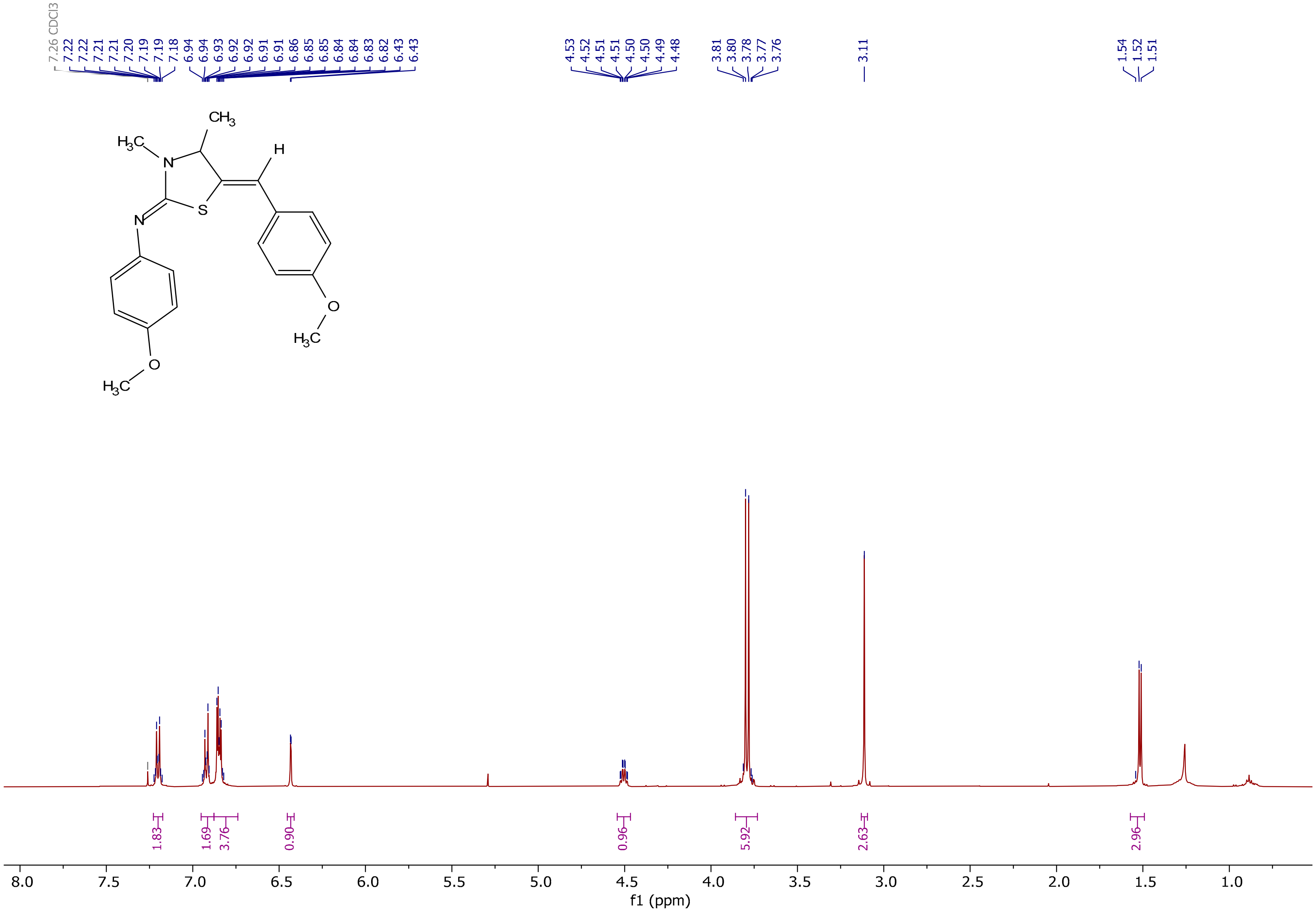

7.26 CDCl3
7.22
7.22
7.21
7.21
7.20
7.19
7.19
7.18
6.94
6.94
6.93
6.92
6.92
6.91
6.91
6.86
6.85
6.85
6.84
6.84
6.83
6.82
6.43
6.43
4.53
4.52
4.51
4.51
4.50
4.50
4.49
4.48
3.81
3.80
3.78
3.77
3.76
3.11
1.54
1.52
1.51
CH3
H3C
N
H
S
N
O
H3C
O
H3C
1.83
1.69
3.76
0.90
0.96
5.92
2.63
2.96
8.0
7.5
7.0
6.5
6.0
5.5
5.0
4.5
4.0
3.5
3.0
2.5
2.0
1.5
1.0
f1 (ppm)

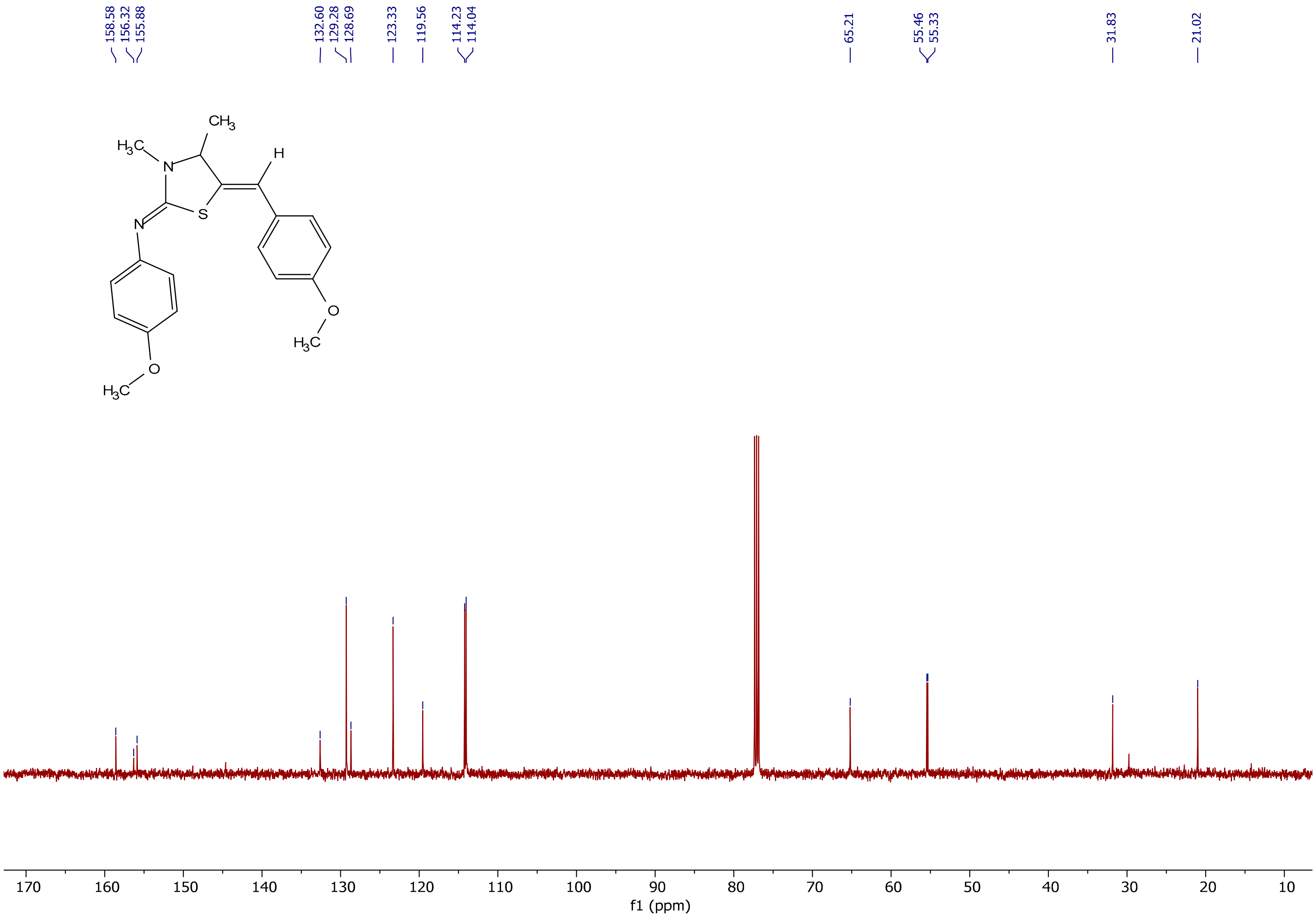

158.58
156.32
155.88
132.60
129.28
128.69
123.33
119.56
114.23
114.04
65.21
55.46
55.33
31.83
21.02
CH3
H3C
N
H
S
N
O
H3C
O
H3C
170
160
150
140
130
120
110
100
90
80
70
60
50
40
30
20
10
f1 (ppm)

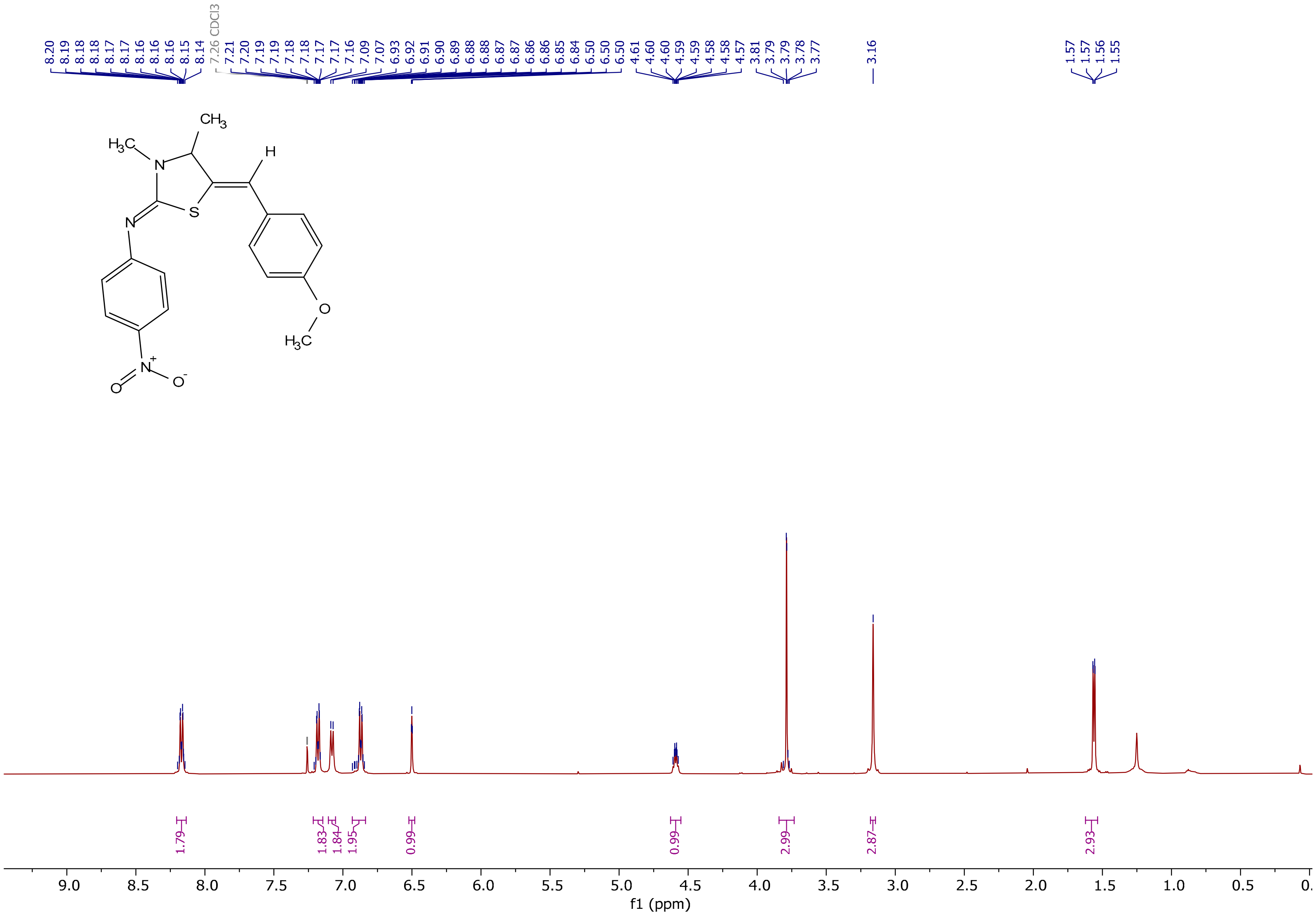

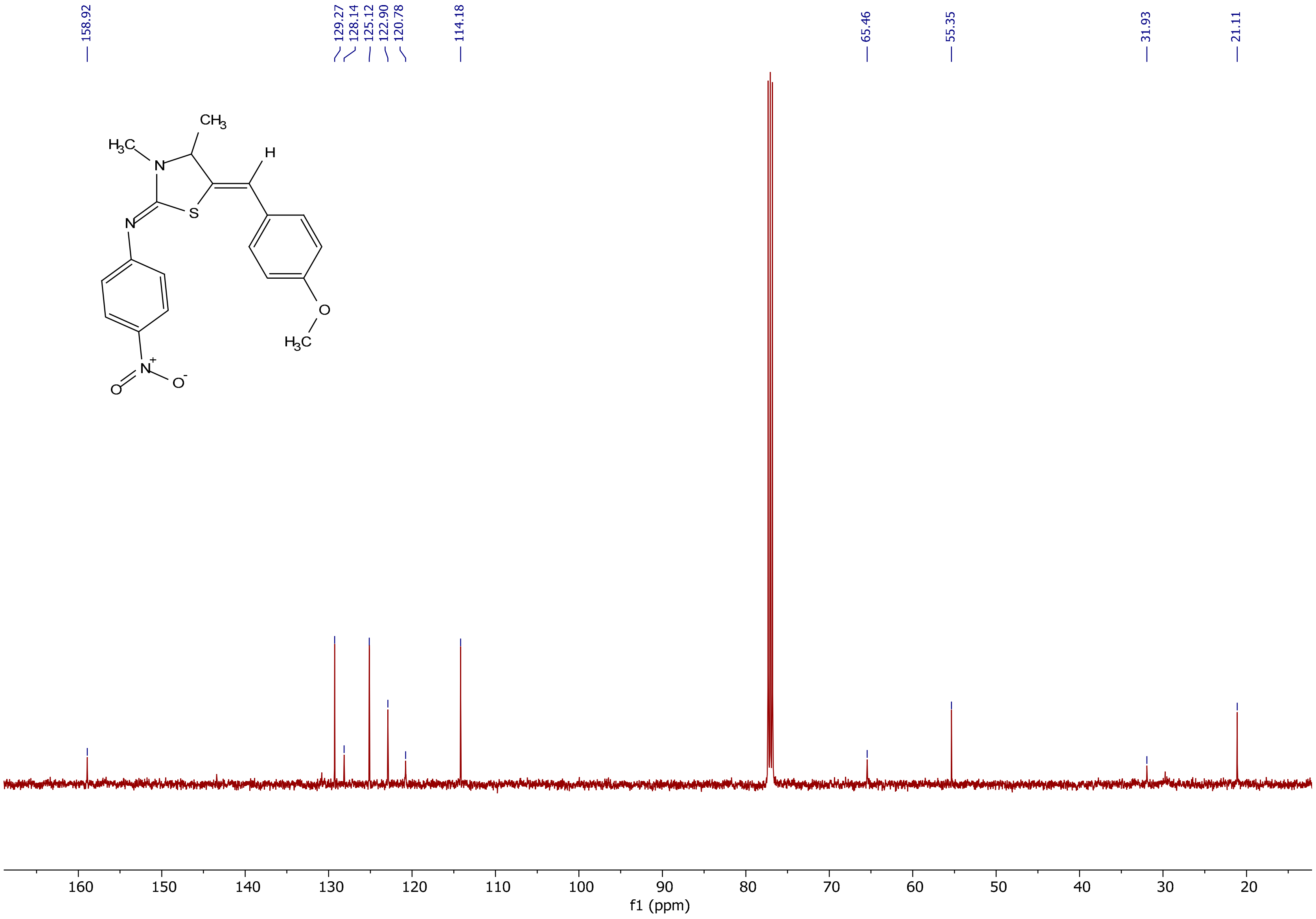

158.92
129.27
128.14
125.12
122.90
120.78
114.18
65.46
55.35
31.93
21.11
CH3
H3C
N
H
S
N
N+
O
O-
H3C
O
160
150
140
130
120
110
100
90
80
70
60
50
40
30
20
f1 (ppm)

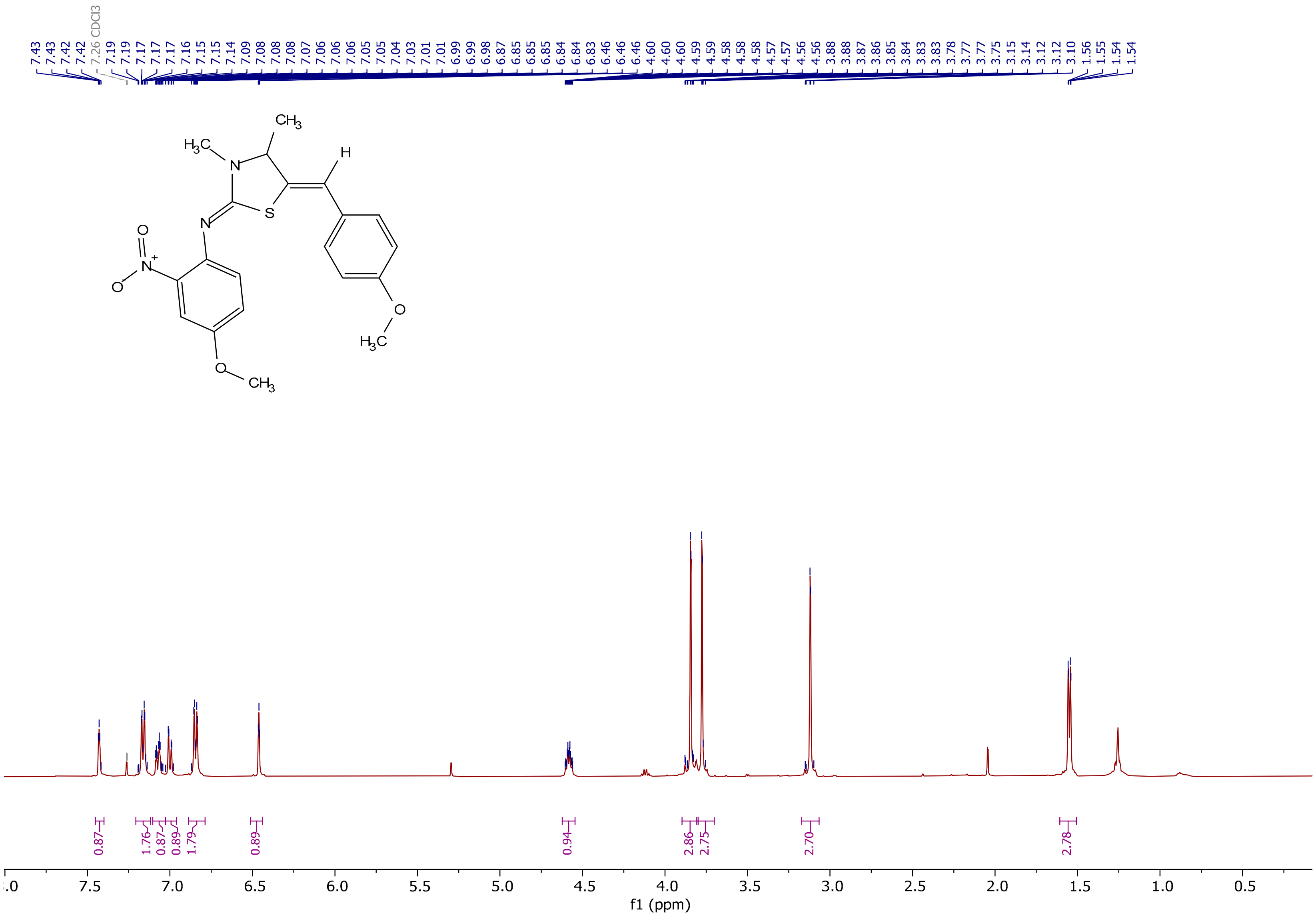
7.43
7.43
7.42
7.26 CDCl3
7.19
7.19
7.17
7.17
7.17
7.16
7.15
7.15
7.14
7.09
7.08
7.08
7.08
7.07
7.06
7.06
7.05
7.05
7.04
7.03
7.01
6.99
6.99
6.98
6.87
6.85
6.85
6.85
6.84
6.84
6.83
6.46
6.46
6.46
4.60
4.60
4.59
4.59
4.58
4.58
4.57
4.57
4.56
4.56
3.88
3.88
3.87
3.86
3.85
3.84
3.83
3.83
3.78
3.77
3.77
3.75
3.15
3.14
3.12
3.12
3.10
1.56
1.55
1.54
1.54
CH3
H3C
N
H
S
N
O
N+
O
O
H3C
O
CH3
0.87
1.76
0.87
0.89
1.79
0.89
0.94
2.86
2.75
2.70
2.78
7.5
7.0
6.5
6.0
5.5
5.0
4.5
4.0
3.5
3.0
2.5
2.0
1.5
1.0
0.5
f1 (ppm)

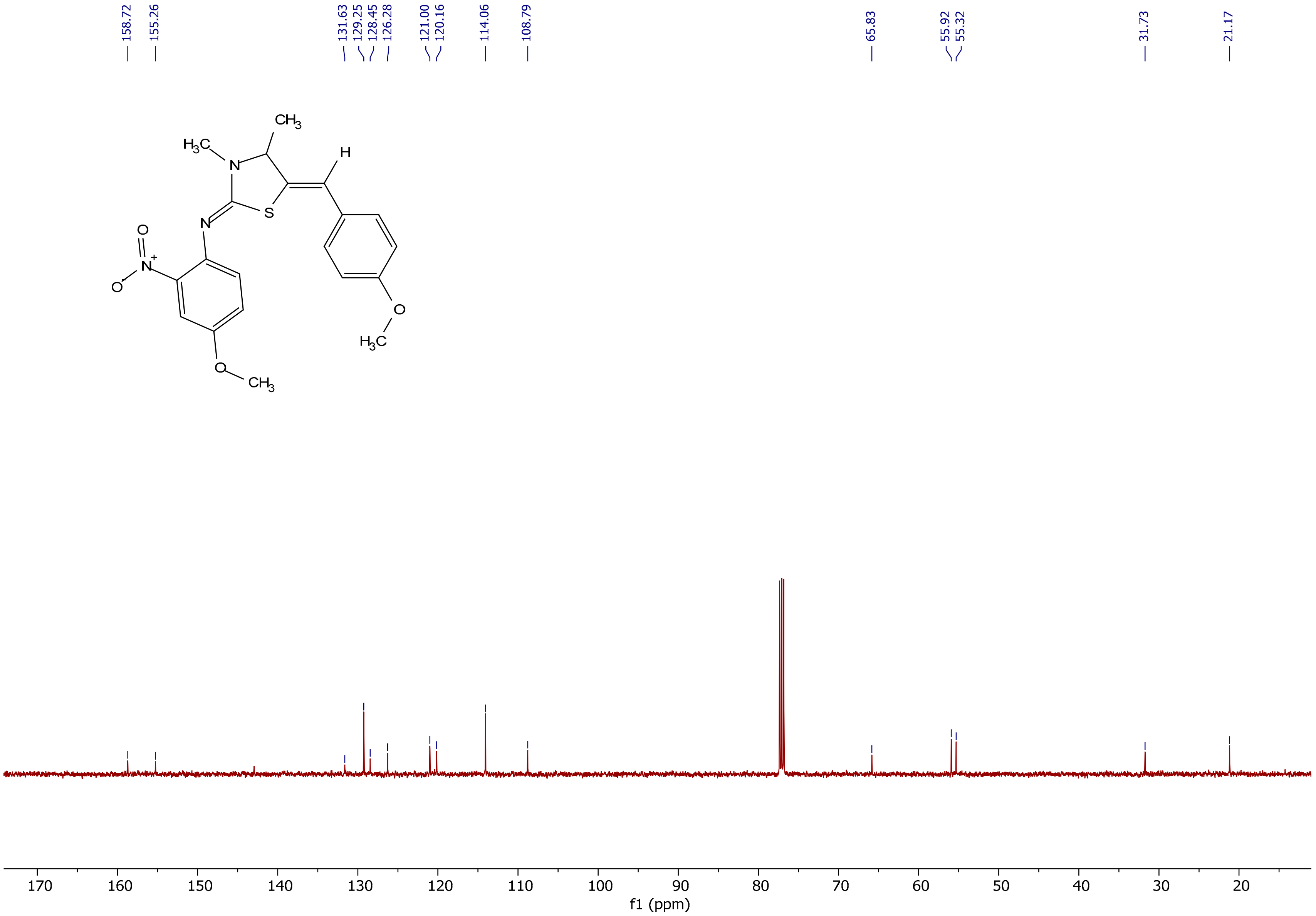
158.72
155.26
131.63
129.25
128.45
126.28
121.00
120.16
114.06
108.79
65.83
55.92
55.32
31.73
21.17
170
160
150
140
130
120
110
100
90
80
70
60
50
40
30
20
f1 (ppm)

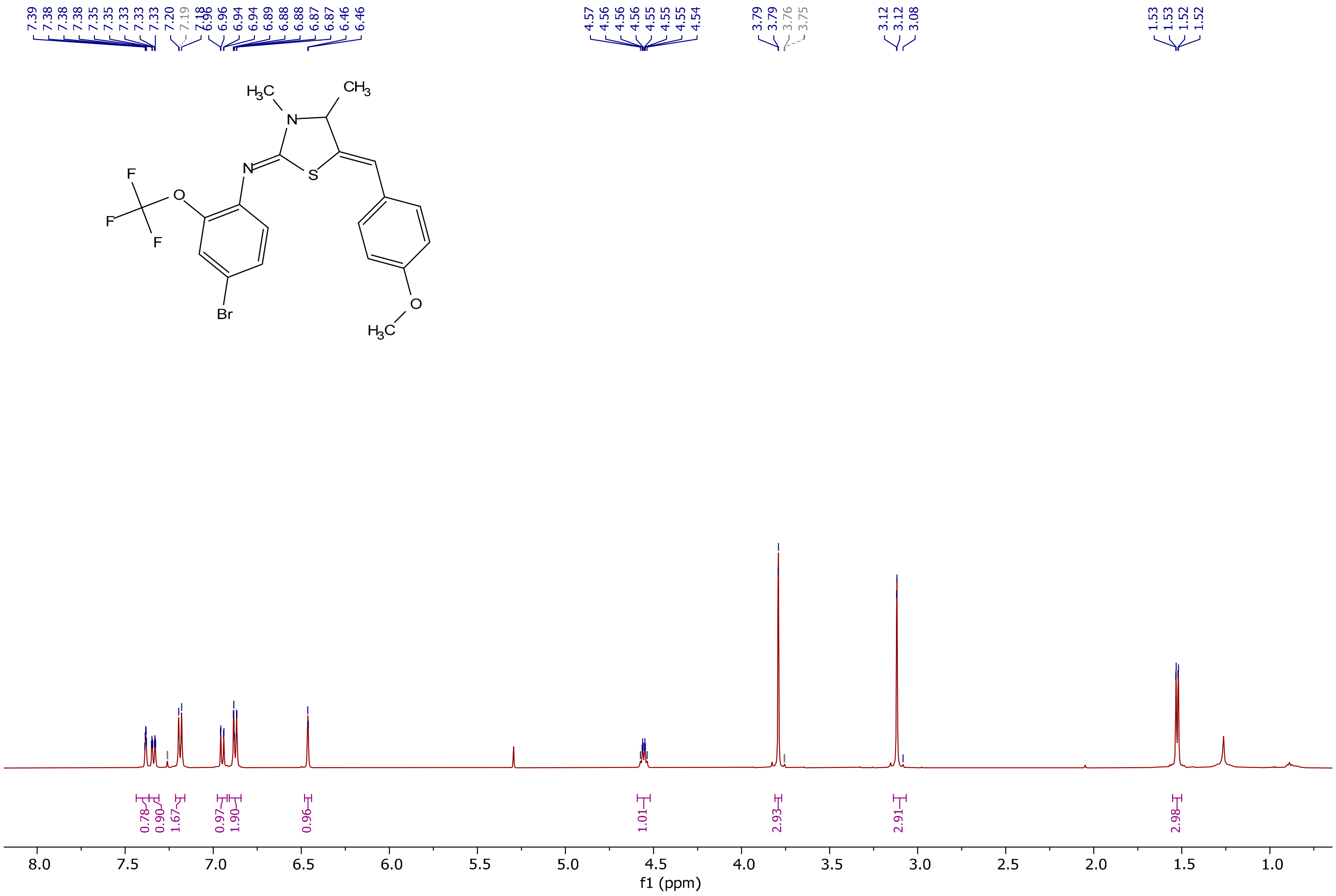

7.39
7.38
7.38
7.38
7.35
7.35
7.33
7.33
7.33
7.33
7.20
7.19
7.18
6.96
6.96
6.94
6.94
6.89
6.88
6.88
6.87
6.87
6.46
6.46
4.57
4.56
4.56
4.56
4.55
4.55
4.55
4.54
3.79
3.79
3.76
3.75
3.12
3.12
3.08
1.53
1.53
1.52
1.52
H3C
CH3
N
N
S
O
F
F
F
Br
O
H3C
0.78
0.90
1.67
0.97
1.90
0.96
1.01
2.93
2.91
2.98
8.0
7.5
7.0
6.5
6.0
5.5
5.0
4.5
4.0
3.5
3.0
2.5
2.0
1.5
1.0
f1 (ppm)

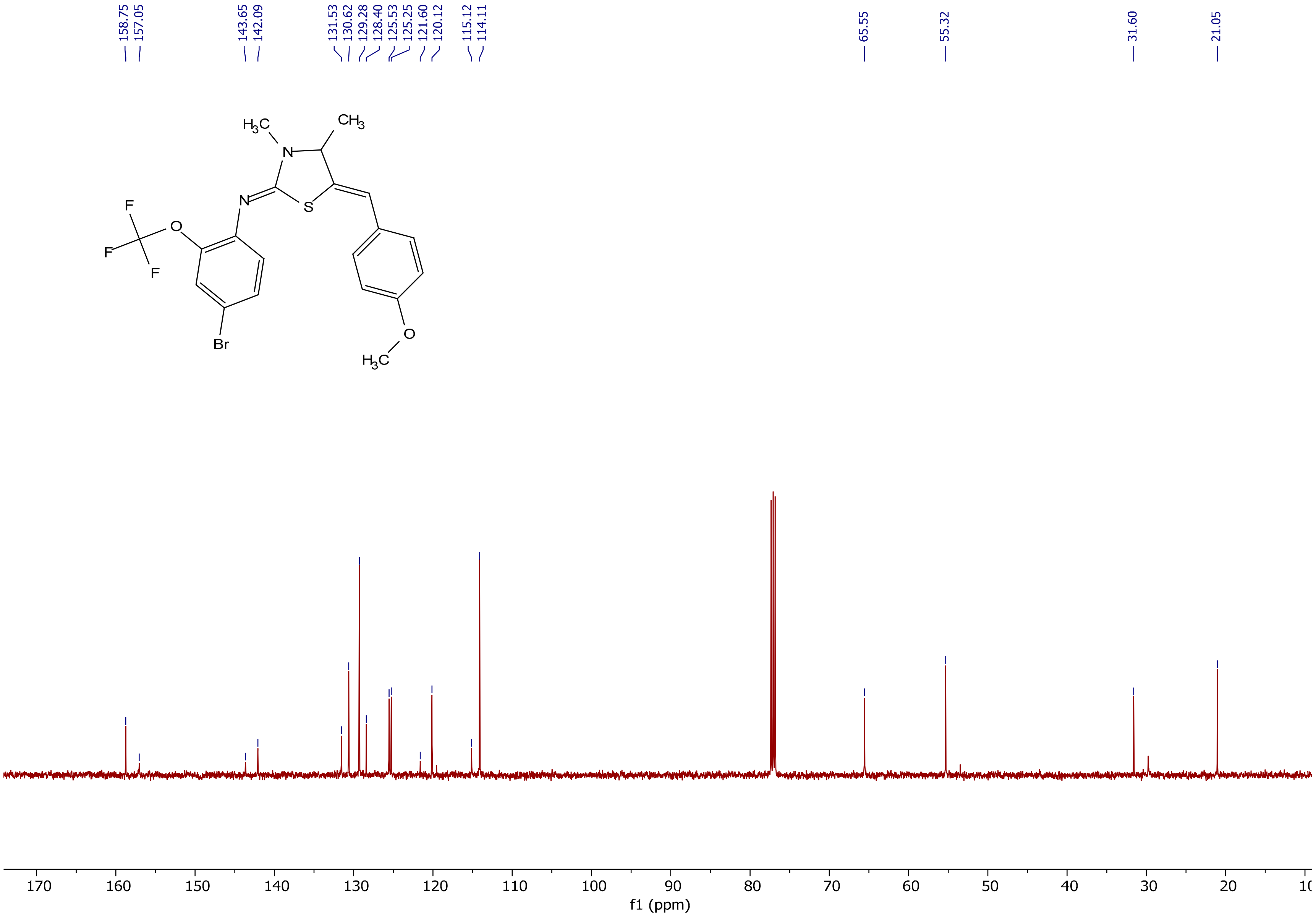
158.75
157.05
143.65
142.09
131.53
130.62
129.28
128.40
125.53
125.25
121.60
120.12
115.12
114.11
65.55
55.32
31.60
21.05
H3C
CH3
N
S
F
O
F
F
Br
O
H3C
170 160 150 140 130 120 110 100 90 80 70 60 50 40 30 20 10
f1 (ppm)
